# Supplementary material for: Inference procedures in sequential trial emulation with survival outcomes: Comparing confidence intervals based on the sandwich variance estimator, bootstrap and jackknife
Source: Stat Methods Med Res. 2025 Jul 9;34(10):2011–33. doi: 10.1177/09622802251356594 (PMC12541114; doi:10.1177/09622802251356594)
Supplement: sj-pdf-1-smm-10.1177_09622802251356594 - Supplemental material for Inference procedures in sequential trial emulation with survival outcomes: Comparing confidence intervals based on the sandwich variance estimator, bootstrap and jackknife [file sj-pdf-1-smm-10.1177_09622802251356594.pdf]

Supplementary Materials to "Inference procedures in sequential trial emulation with survival outcomes: comparing confidence intervals based on the sandwich variance estimator, bootstrap and jackknife"

1 Schematic illustration of sequential trial emulation

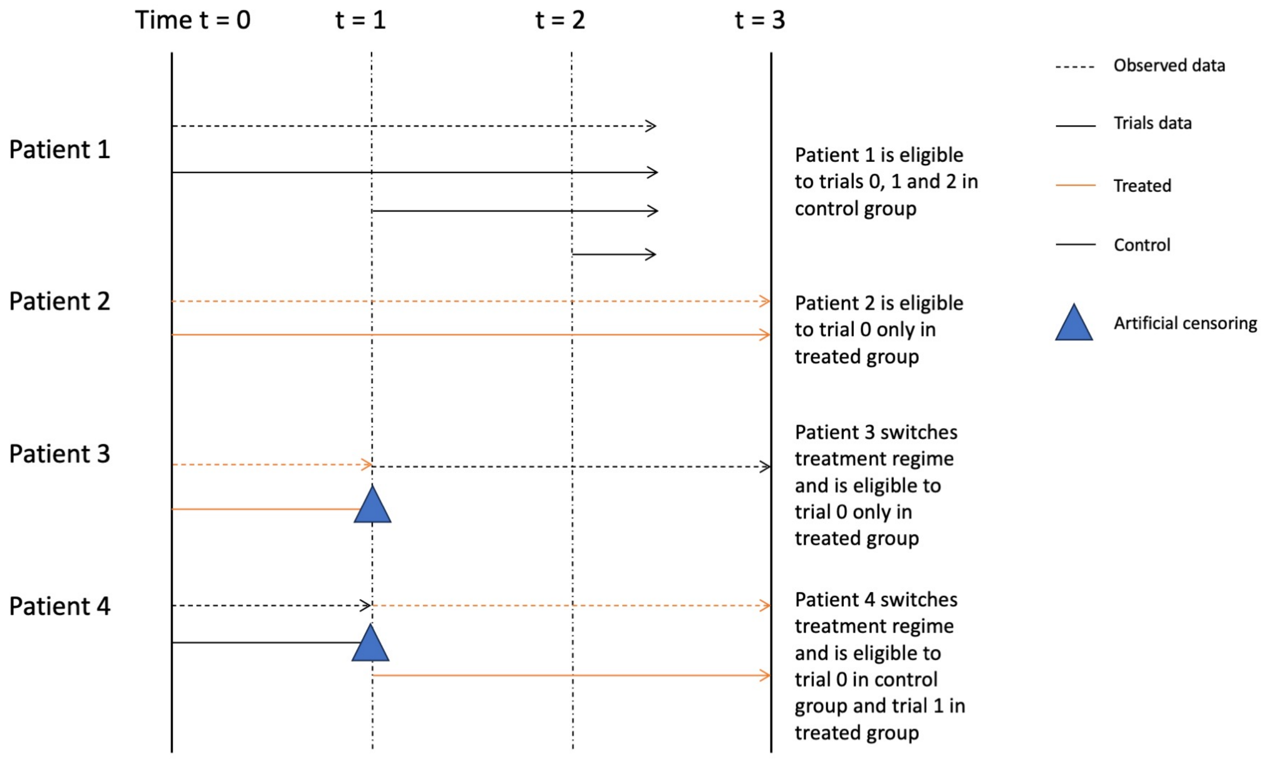

Figure 1: A schematic illustration of how patients' data are utilised in sequential trial emulation.

Figure 1 provides a schematic illustration of how patients' data are utilised in sequential trial emulation (STE). Three target trials, starting at visits at time $t = 0, 1, 2$, were sequentially emulated, which were referred as 'trial 0', 'trial 1', and 'trial 2'. Patient 1 never initiated treatment for the duration of the study until he/she was lost to follow-up after $t = 2$, so he/she/they was eligible as a control (marked by the black arrow) in all three

trials. Patient 2 initiated treatment (marked by the orange arrow) at $t = 0$ and kept taking the treatment until the end of follow-up so he/she/they was in the treated group for trial 0 starting at $t = 0$, but not eligible for trials 1 and 2, starting at $t = 1, 2$, respectively, under the criterion that patients were only eligible if there was no prior history of treatment. Patient 3 initiated treatment at $t = 0$ but stopped from $t = 1$, so his/her/their follow-up was artificially censored from $t = 1$. Patient 3 was included in the treatment group for trial 0, but his/her data was not included in other trials because he/she/they had a prior history of treatment. Patient 4 was not taking treatment at $t = 0$ but initiated treatment from $t = 1$ onwards: hence he/she/they was eligible as a control in trial 0 and artificially censored at the time he/she started treatment, $t = 1$, and he/she was eligible as a treated patient in trial 1 but not eligible in trial 2.

2 Inverse probability weighting

For addressing artificial censoring due to treatment non-adherence, a patient's *stabilised inverse probability of treatment weight* [1, 2, 3, 4] at trial visit k ($k > 0$) in trial m is defined as

$$sw^A_{m,k} = \frac{\prod_{j=1}^{k} \Pr(A_{m,j} = a \mid \overline{A}_{m,j-1} = \overline{a}_{j-1}, \boldsymbol{V}, \boldsymbol{L}_{m,0}, E_m = 1, Y_{m,j-1} = 0, C_{m,j-1} = 0)}{\prod_{j=1}^{k} \Pr(A_{m,j} = a \mid \overline{A}_{m,j-1} = \overline{a}_{j-1}, \boldsymbol{V}, \overline{\boldsymbol{L}}_{m,j}, E_m = 1, Y_{m,j-1} = 0, C_{m,j-1} = 0)}, \qquad a \in \{0, 1\}. \tag{1}$$

Here, $\Pr(A_{m,j} = a \mid \overline{A}_{m,j-1} = \overline{a}_{j-1}, \boldsymbol{V}, \overline{\boldsymbol{L}}_{m,j}, E_m = 1, Y_{m,j-1} = 0, C_{m,j-1} = 0)$ is the conditional probability that the patient's treatment received at trial visit j in trial m remained the same as their treatment received up to trial visit $j - 1$, conditional on their observed variables up to trial visit j. The baseline covariates $\boldsymbol{V}$ and $\boldsymbol{L}_{m,0}$ in the numerator of (1) are also included as covariates in the MSM of equation (3) of the main text [2].

To address censoring due to loss to follow-up, the *stabilised inverse probability of censoring weight* [1, 2, 3, 4] for a patient at trial visit k ($k > 0$) in trial m is

$$sw^C_{m,k} = \frac{\prod_{j=0}^{k-1} \Pr(C_{m,j} = 0 \mid C_{m,j-1} = 0, \overline{A}_{m,j} = \overline{a}_j, \boldsymbol{V}, \boldsymbol{L}_{m,0}, E_m = 1, Y_{m,j} = 0)}{\Pi_{j=0}^{k-1} \Pr(C_{m,j} = 0 \mid C_{m,j-1} = 0, \overline{A}_{m,j} = \overline{a}_j, \boldsymbol{V}, \overline{\boldsymbol{L}}_{m,j}, E_m = 1, Y_{m,j} = 0)}, \qquad a \in \{0, 1\}. \tag{2}$$

Here, $\Pr(C_{m,j} = 0 \mid \overline{C}_{m,j-1} = 0, \overline{A}_{m,j} = \overline{a}_j, \boldsymbol{V}, \overline{\boldsymbol{L}}_{m,j}, E_m = 1, Y_{m,j} = 0)$ is the conditional probability that the patient remains in the trial during $[t_{m,j+1}, t_{m,j+2})$ given that they had been in the trial in $[0, t_{m,j+1})$ and their observed covariate history up to time $t_{m,j}$. Again, whenever we condition on certain variables in the numerator of (2), these variables are also be included as covariates in the MSM of equation (3) of the main text.

3 More details for key functions of the `TrialEmulation` package

- `data_preparation`: takes longitudinal data and the specifications of the treatment and censoring models as input, and returns an expanded dataset with patients' data spread across their eligible sequentially

emulated trials. This dataset includes a column containing the patients' SIPTCWs as calculated from the formulae (4) and (5), according to whether we are conducting an intention-to-treat or per-protocol analysis. The modelling fitting required for calculating SIPTCWs was performed using the original longitudinal data with the variables specified in the function input. For the per-protocol analysis, the expanded data have been artificially censored whenever patients stop adhering to their assigned treatment strategies in each emulated trial. This function implements Steps 1 and 2 of MRD estimation described at the end of Section 3 of the main text.

- `trial_msm`: takes the expanded data with SIPTCWs such as one returned from `data_preparation` and the MSM specification such as in equation (3) in the main text as inputs and produces a `fit.glm` type output of the MSM with estimated hazard ratios and the sandwich variance matrix estimate. This function implements Step 3 of MRD estimation described at the end of Section 3 of the main text. We can use `predict.glm` on the output of this function to estimate marginal risks for a target population with given baseline covariate data, which is what we use to carry out Step 5 of the MRD estimation.

- `initiators`: a wrapper function combining the two functions above.

The `data_preparation` function in the `TrialEmulation` package was used to estimate the stabilised IPTWs from the simulated datasets, and this function outputs the expanded and artificially censored data in the sequential trials as described in Section 3 of the main text, with each patient's inverse probability weights at each trial's follow-up visit. The MSM for the discrete-time counterfactual hazard was fitted using the `trial_msm` function.

The sandwich variance matrix estimate is provided in the output of `trial_msm` and was used to obtain Sandwich CIs. The code for implementing nonparametric bootstrap CIs, LEF bootstrap CIs and jackknife CIs, were not included in the `TrialEmulation` package so they were created for the purpose of this article. More details on the `TrialEmulation` package can be found at `https://cran.r-project.org/web/packages/TrialEmulation/TrialEmulation.pdf`

4 Pseudo code for the simulation study

Algorithm 1 presents a pseudo-code of the simulation study in Section 5 of the main text.

Algorithm 1 Pseudo-code of Monte Carlo simulation algorithm.

for MC iteration $m = 1, 2, ..., 1000$ **do**
  for Simulation scenario $l = 1, 2, ..., 81$ **do**
    Data A ← Generate data with scenario l
    Select eligible patients
    Fit models for the denominator and numerator terms of stabilised IPTWs to eligible patients' data up to the first visit they switched treatment after trial baseline.
    Data B ← the original dataset expanded into data for sequentially emulated trials and assign patients to eligible trials
    for Each trial **do**
      Artificially censor patients' follow-up when they no longer adhere to the treatment assigned at the trial baseline
      Estimate each patient's stabilised IPTW based on covariate history in this trial. At trial baseline, the weights are always one.
    end for
    Fit weighted pooled logistic regression to Data B
    v ← MRD estimate for trial 0 patients
    $[B_1, B_2]$ ← lower- and upper-bound of nonparametric bootstrap pivot CI of MRD
    $[LEF_1^{(1)}, LEF_2^{(1)}]$ ← lower- and upper-bound of LEF bootstrap pivot CI of MRD using approach 1
    $[LEF_1^{(2)}, LEF_2^{(2)}]$ ← lower- and upper-bound of LEF bootstrap pivot CI of MRD using approach 2
    $[S_1, S_2]$ ← lower- and upper-bound of the sandwich-variance-estimator-based CI of MRD
    $[J_1^{(1)}, J_2^{(1)}]$ ← lower- and upper-bound of Jackknife Wald CI of MRD
    $[J_1^{(2)}, J_2^{(2)}]$ ← lower- and upper-bound of jackknife MVN CI of MRD
  end for
end for
return $v, [B_1, B_2], [LEF_1^{(1)}, LEF_2^{(1)}], [LEF_1^{(2)}, LEF_2^{(2)}], [S_1, S_2], [J_1^{(1)}, J_2^{(1)}], [J_1^{(2)}, J_2^{(2)}]$

5 Additional simulation results

5.1 Data tabulation of sequentially emulated trials in simulations

Tables 1–9 provide examples of data prepared for sequentially emulated trials using simulated data in Section 3 of the main text. Data are aggregated across trials and tabulated by treatment arm, trial visit and outcome status in various simulation scenarios.

Table 1: Data tabulation by treatment arm and outcome status when generating one dataset using the data generating algorithm in Table 2 of the Main Text for **small sample sizes** ($n = 200$) **under low event rate**.

					Trial visit				
Sample size	Confounding strength	Treatment prevalence	Assigned treatment	Outcome	0	1	2	3	4
200	0.1	-1	0	0	409	269	172	98	43
				1	5	4	1	0	0
			1	0	148	34	11	2	1
				1	4	2	0	0	0
		0	0	0	162	77	37	16	5
				1	5	2	1	0	0
			1	0	190	89	45	25	7
				1	0	0	0	1	0
		1	0	0	85	29	8	3	1
				1	0	0	0	0	0
			1	0	198	149	108	74	42
				1	1	2	0	0	0
	0.5	-1	0	0	400	254	156	84	34
				1	3	2	0	0	0
			1	0	162	40	11	2	0
				1	1	2	1	0	0
		0	0	0	200	98	47	21	8
				1	2	1	0	0	0
			1	0	187	101	47	24	12
				1	3	0	1	0	0
		1	0	0	81	29	11	5	2
				1	0	0	0	0	0
			1	0	196	130	95	64	36
				1	2	1	0	2	0
	0.9	-1	0	0	397	257	156	86	37
				1	1	1	0	0	0
			1	0	160	34	11	2	0
				1	2	1	0	0	0
		0	0	0	194	99	49	25	10
				1	2	2	1	0	0
			1	0	185	80	36	16	5
				1	3	2	1	0	0
		1	0	0	97	30	9	4	2
				1	0	0	0	0	0
			1	0	197	150	97	58	28
				1	1	2	1	0	0

Table 2: Data tabulation by treatment arm and outcome status when generating one dataset using the data generating algorithm in Table 2 of the Main Text for **medium sample sizes** ($n = 1000$) **under low event rate**.

Sample size	Confounding strength	Treatment prevalence	Assigned treatment	Outcome	Trial visit 0	1	2	3	4
1000	0.1	-1	0	0	2058	1357	839	462	193
				1	35	24	16	9	5
			1	0	765	188	41	7	2
				1	7	2	2	0	0
		0	0	0	952	480	239	111	42
				1	13	6	3	0	0
			1	0	937	473	231	91	42
				1	8	5	3	2	0
		1	0	0	357	81	13	2	0
				1	6	0	0	0	0
			1	0	991	727	536	358	222
				1	3	7	5	5	0
	0.5	-1	0	0	1960	1265	758	412	173
				1	27	20	10	6	1
			1	0	790	196	45	10	2
				1	10	5	0	0	0
		0	0	0	977	464	211	83	29
				1	12	6	2	2	1
			1	0	951	450	183	82	26
				1	8	4	4	0	1
		1	0	0	398	114	31	10	2
				1	6	2	1	0	0
			1	0	978	687	462	304	168
				1	14	3	6	2	2
	0.9	-1	0	0	1897	1215	730	394	161
				1	24	9	8	4	0
			1	0	799	189	49	8	1
				1	16	2	0	0	0
		0	0	0	968	475	219	83	26
				1	17	6	2	0	0
			1	0	939	414	170	68	19
				1	18	7	1	1	0
		1	0	0	436	134	43	13	2
				1	0	0	0	0	0
			1	0	987	633	406	250	131
				1	11	5	3	8	2

Table 3: Data tabulation by treatment arms and outcome status when generating one dataset using the data generating algorithm in Table 2 of the Main Text for **large sample sizes** ($n = 5000$) **under low event rate**.

					Trial visit				
Sample size	Confounding strength	Treatment prevalence	Assigned treatment	Outcome	0	1	2	3	4
5000	0.1	-1	0	0	10181	6601	4075	2273	961
				1	119	70	43	23	13
			1	0	3891	976	250	49	12
				1	29	6	2	2	1
		0	0	0	4949	2446	1171	522	177
				1	52	22	12	8	3
			1	0	4732	2251	1052	477	145
				1	39	18	13	2	2
		1	0	0	1932	557	168	47	11
				1	27	6	1	0	0
			1	0	4912	3531	2545	1797	1028
				1	50	20	31	11	14
	0.5	-1	0	0	10134	6539	3992	2193	916
				1	135	85	50	24	11
			1	0	3902	934	201	36	3
				1	47	5	4	0	0
		0	0	0	4858	2388	1144	517	185
				1	65	32	16	6	2
			1	0	4698	2151	973	406	127
				1	52	16	17	7	5
		1	0	0	1931	545	153	42	10
				1	30	6	2	0	0
			1	0	4911	3426	2365	1568	865
				1	49	30	20	17	14
	0.9	-1	0	0	9673	6170	3729	2051	842
				1	151	100	60	37	17
			1	0	3922	925	205	50	10
				1	85	10	2	0	0
		0	0	0	4695	2267	1050	462	151
				1	55	27	9	4	1
			1	0	4727	1986	846	336	104
				1	67	29	9	7	0
		1	0	0	2183	644	196	53	15
				1	20	7	0	0	0
			1	0	4894	3147	1985	1231	603
				1	71	42	34	18	15

Table 4: Data tabulation by treatment arms and outcome status when generating one dataset using the data generating algorithm in Table 2 of the Main Text for **small sample sizes ($n = 200$) under medium event rate**.

					Trial visit				
Sample size	Confounding strength	Treatment prevalence	Assigned treatment	Outcome	0	1	2	3	4
200	0.1	-1	0	0	391	253	157	87	36
				1	12	8	4	3	1
			1	0	150	32	7	1	0
				1	2	1	0	0	0
		0	0	0	183	89	47	22	8
				1	8	6	2	2	0
			1	0	179	87	40	14	6
				1	5	4	1	0	0
		1	0	0	87	32	13	4	1
				1	2	0	0	0	0
			1	0	191	138	102	71	46
				1	6	3	1	1	1
	0.5	-1	0	0	417	276	177	100	44
				1	14	8	4	3	0
			1	0	139	29	7	2	0
				1	3	0	0	1	0
		0	0	0	196	102	53	29	11
				1	4	3	2	0	0
			1	0	179	82	25	8	0
				1	6	0	2	0	0
		1	0	0	70	18	5	1	0
				1	2	0	0	0	0
			1	0	195	134	104	71	37
				1	3	4	0	1	0
	0.9	-1	0	0	357	226	136	72	32
				1	6	3	3	2	0
			1	0	156	46	8	2	1
				1	6	4	1	0	0
		0	0	0	178	79	36	13	3
				1	5	4	0	0	0
			1	0	181	80	34	14	2
				1	11	3	4	0	0
		1	0	0	81	27	9	4	0
				1	1	0	0	0	0
			1	0	190	122	80	41	21
				1	9	5	3	1	1

Table 5: Data tabulation by treatment arms and outcome status when generating one dataset using the data generating algorithm in Table 2 of the Main Text for **medium sample sizes** (n = 1000) **under medium event rate**.

Sample size	Confounding strength	Treatment prevalence	Assigned treatment	Outcome	Trial visit 0	1	2	3	4
1000	0.1	-1	0	0	2012	1321	825	460	194
				1	66	40	20	11	3
			1	0	722	176	37	6	1
				1	18	8	1	0	0
		0	0	0	1002	505	257	127	44
				1	32	17	5	1	0
			1	0	902	412	179	73	25
				1	22	8	4	0	0
		1	0	0	360	86	20	4	1
				1	9	3	1	0	0
			1	0	972	669	453	312	182
				1	18	19	10	9	5
	0.5	-1	0	0	1997	1275	772	417	170
				1	52	28	16	9	1
			1	0	752	172	37	10	2
				1	26	5	3	0	0
		0	0	0	997	506	249	113	37
				1	22	9	3	2	2
			1	0	913	397	173	76	31
				1	28	11	0	3	2
		1	0	0	402	117	31	6	1
				1	16	2	1	0	0
			1	0	951	642	423	272	144
				1	32	16	11	5	5
	0.9	-1	0	0	1742	1093	662	363	151
				1	65	41	21	13	5
			1	0	755	179	48	11	3
				1	29	11	1	1	0
		0	0	0	937	453	223	108	41
				1	16	7	3	1	1
			1	0	905	409	177	56	13
				1	38	12	5	2	0
		1	0	0	409	113	33	6	1
				1	9	2	0	0	0
			1	0	952	608	395	236	121
				1	38	16	15	9	6

Table 6: Data tabulation by treatment arms and outcome status when generating one dataset using the data generating algorithm in Table 2 of the Main Text for **large sample sizes** ($n = 5000$) **under medium event rate**.

					Trial visit				
Sample size	Confounding strength	Treatment prevalence	Assigned treatment	Outcome	0	1	2	3	4
5000	0.1	-1	0	0	9935	6440	3976	2209	934
				1	298	178	98	49	16
			1	0	3679	911	215	42	7
				1	89	24	7	1	0
		0	0	0	4736	2298	1076	472	161
				1	127	48	22	13	3
			1	0	4624	2257	1037	445	132
				1	88	55	18	15	1
		1	0	0	1741	454	125	35	7
				1	59	10	2	0	0
			1	0	4826	3458	2427	1645	913
				1	108	58	61	40	29
	0.5	-1	0	0	9471	6047	3655	1988	809
				1	299	180	117	57	26
			1	0	3785	904	224	48	5
				1	107	21	6	1	1
		0	0	0	4727	2298	1077	477	164
				1	129	50	30	11	3
			1	0	4570	2069	906	359	125
				1	137	47	25	12	3
		1	0	0	1846	497	124	32	9
				1	47	8	2	0	0
			1	0	4820	3247	2179	1380	748
				1	124	64	45	32	20
	0.9	-1	0	0	8950	5602	3328	1769	710
				1	297	165	93	55	24
			1	0	3813	907	201	38	7
				1	180	30	6	3	3
		0	0	0	4795	2318	1124	485	166
				1	124	47	19	7	1
			1	0	4547	1917	805	304	84
				1	163	80	14	13	8
		1	0	0	2041	615	192	58	16
				1	47	10	6	3	1
			1	0	4775	3030	1941	1185	592
				1	162	81	50	41	20

Table 7: Data tabulation by treatment arms and outcome status when generating one dataset using the data generating algorithm in Table 2 of the Main Text for **small sample sizes ($n = 200$) under high event rate**.

Sample size	Confounding strength	Treatment prevalence	Assigned treatment	Outcome	Trial visit 0	1	2	3	4
200	0.1	-1	0	0	345	213	126	67	28
				1	17	13	7	4	1
			1	0	148	32	14	3	1
				1	7	2	1	1	0
		0	0	0	187	95	46	20	5
				1	12	2	2	0	0
			1	0	180	83	33	14	5
				1	3	5	2	2	1
		1	0	0	71	20	6	1	0
				1	3	0	0	0	0
			1	0	188	121	83	49	29
				1	9	8	4	3	1
	0.5	-1	0	0	354	226	140	80	34
				1	23	14	9	5	2
			1	0	135	31	5	2	0
				1	8	1	1	0	0
		0	0	0	192	96	45	19	7
				1	13	4	1	1	0
			1	0	173	81	36	7	2
				1	7	4	1	1	0
		1	0	0	55	12	5	1	0
				1	1	0	0	0	0
			1	0	188	127	82	55	36
				1	11	9	4	3	0
	0.9	-1	0	0	377	238	145	75	31
				1	24	14	8	6	4
			1	0	136	24	6	1	0
				1	9	1	0	0	0
		0	0	0	187	91	46	20	6
				1	10	4	3	2	0
			1	0	172	72	30	6	2
				1	12	2	2	3	0
		1	0	0	74	18	0	0	0
				1	4	2	1	0	0
			1	0	188	125	74	49	24
				1	8	10	6	1	0

Table 8: Data tabulation by treatment arms and outcome status when generating one dataset using the data generating algorithm in Table 2 of the Main Text for **medium sample sizes ($n = 1000$) under high event rate**.

					Trial visit				
Sample size	Confounding strength	Treatment prevalence	Assigned treatment	Outcome	0	1	2	3	4
1000	0.1	-1	0	0	1865	1198	722	393	165
				1	105	55	33	18	7
			1	0	695	154	27	6	0
				1	35	6	1	0	0
		0	0	0	802	373	167	69	23
				1	42	11	7	2	1
			1	0	894	421	199	95	41
				1	41	10	9	5	1
		1	0	0	365	109	33	10	3
				1	22	3	1	0	0
			1	0	925	627	431	277	156
				1	50	29	19	14	10
	0.5	-1	0	0	1871	1178	709	382	158
				1	99	57	30	13	5
			1	0	695	159	34	3	1
				1	48	6	4	0	0
		0	0	0	828	381	169	74	26
				1	43	20	6	4	0
			1	0	885	396	171	62	14
				1	46	16	4	4	1
		1	0	0	346	93	23	5	0
				1	34	11	2	0	0
			1	0	912	582	387	256	126
				1	54	31	7	7	5
	0.9	-1	0	0	1698	1034	612	330	132
				1	105	62	27	14	7
			1	0	712	144	33	11	3
				1	51	18	4	0	0
		0	0	0	899	417	184	79	28
				1	59	27	12	4	0
			1	0	850	357	151	45	15
				1	63	34	3	7	1
		1	0	0	457	155	48	18	7
				1	20	5	2	1	0
			1	0	919	565	355	217	118
				1	54	41	20	11	8

Table 9: Data tabulation by treatment arms and outcome status when generating one dataset using the data generating algorithm in Table 2 of the Main Text for **large sample sizes ($n = 5000$) under high event rate**.

					Trial visit				
Sample size	Confounding strength	Treatment prevalence	Assigned treatment	Outcome	0	1	2	3	4
5000	0.1	-1	0	0	9584	6122	3754	2069	851
				1	593	356	188	85	40
			1	0	3378	763	171	28	5
				1	178	43	4	3	0
		0	0	0	4440	2076	951	395	129
				1	249	111	42	21	1
			1	0	4426	2069	949	395	143
				1	196	80	48	33	7
		1	0	0	1739	471	125	34	9
				1	89	25	10	1	0
			1	0	4687	3258	2277	1508	845
				1	215	146	110	78	35
	0.5	-1	0	0	9292	5884	3571	1958	808
				1	574	341	193	93	39
			1	0	3428	786	170	35	5
				1	190	48	11	2	1
		0	0	0	4482	2112	997	452	157
				1	261	97	45	23	6
			1	0	4382	1972	859	321	105
				1	200	90	35	13	2
		1	0	0	1772	484	131	29	6
				1	106	20	5	1	0
			1	0	4644	3119	2033	1328	746
				1	244	134	122	51	34
	0.9	-1	0	0	8655	5343	3152	1676	672
				1	545	317	172	94	45
			1	0	3451	769	192	42	7
				1	332	56	13	5	2
		0	0	0	4517	2133	971	405	129
				1	256	114	46	17	7
			1	0	4295	1776	704	253	78
				1	320	94	43	20	4
		1	0	0	1920	528	161	44	10
				1	107	19	9	2	0
			1	0	4558	2802	1696	1013	475
				1	325	167	90	54	27

5.2 CI coverage in medium and high event rate scenarios

Figures 2 and 3 present the empirical coverage rates of the CIs when the event rates were medium and high, respectively.

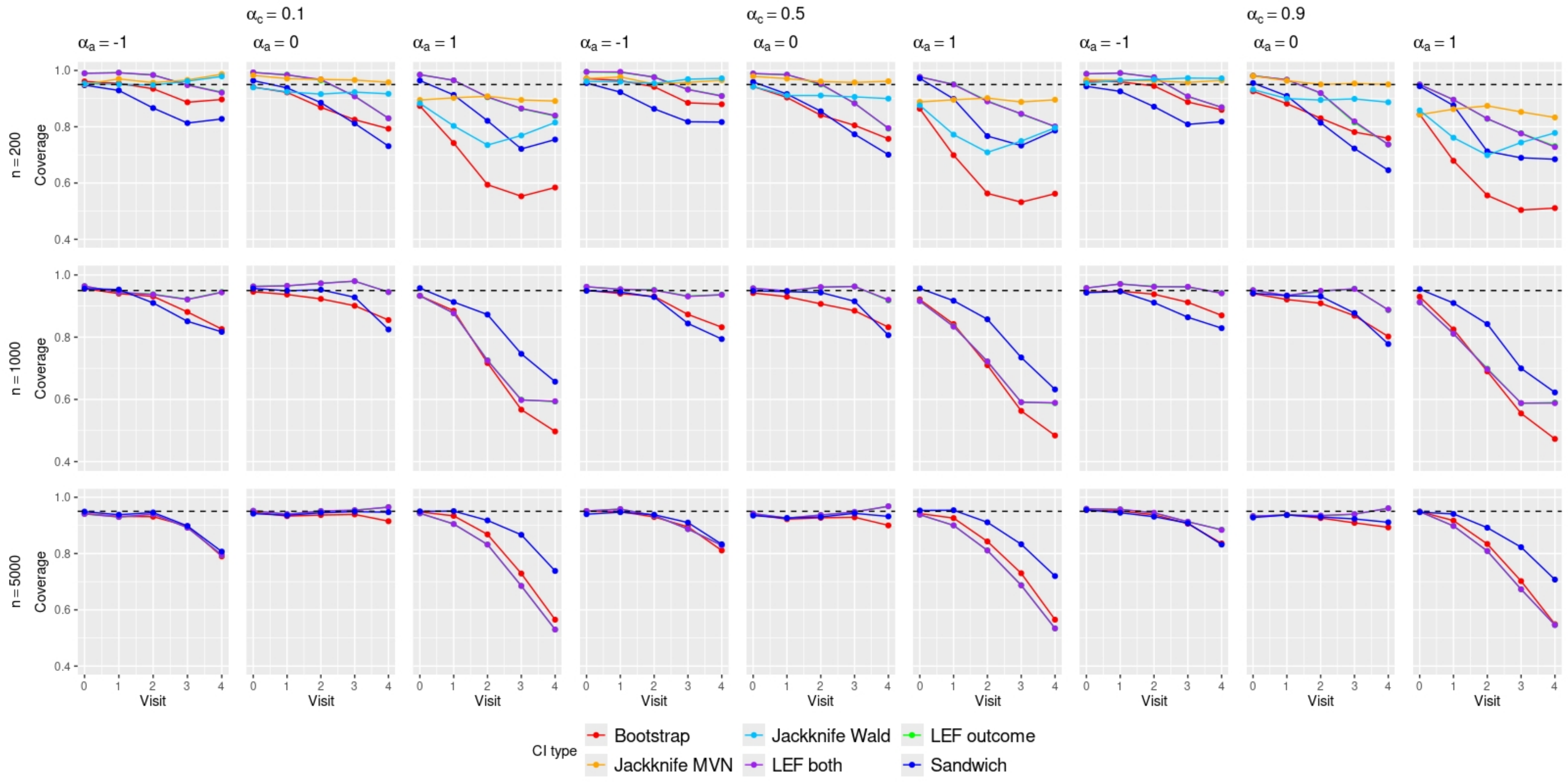

Figure 2: Coverage of the CIs under **medium event rates**. Bootstrap: CIs constructed by nonparametric bootstrap; LEF both: CIs constructed by applying Approach 2 of LEF bootstrap; LEF outcome: CIs constructed by applying Approach 1 of LEF bootstrap; Jackknife Wald: CIs constructed by applying Approach 1 of Jackknife resampling; Jackknife MVN: CIs constructed by applying Approach 2 of Jackknife resampling; Sandwich: CIs based on the sandwich variance estimator

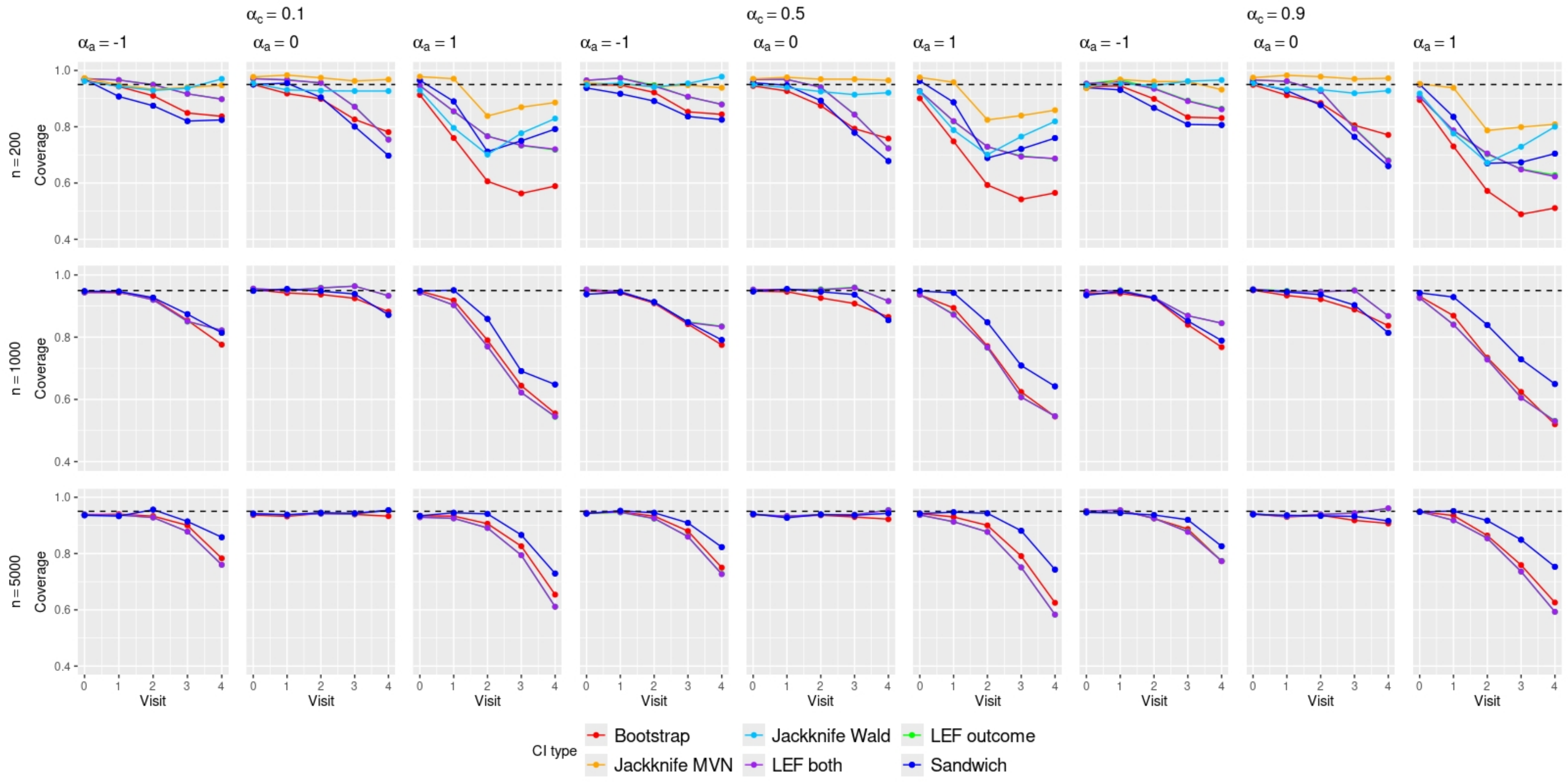

Figure 3: Coverage of the CIs under **high event rates**. Bootstrap: CIs constructed by nonparametric bootstrap; LEF both: CIs constructed by applying Approach 2 of LEF bootstrap; LEF outcome: CIs constructed by applying Approach 1 of LEF bootstrap; Jackknife Wald: CIs constructed by applying Approach 1 of Jackknife resampling; Jackknife MVN: CIs constructed by applying Approach 2 of Jackknife resampling; Sandwich: CIs based on the sandwich variance estimator

5.3 Bias-eliminated CI coverage

Figures 4–6 present bias-eliminated coverage rates under different outcome rate scenarios.

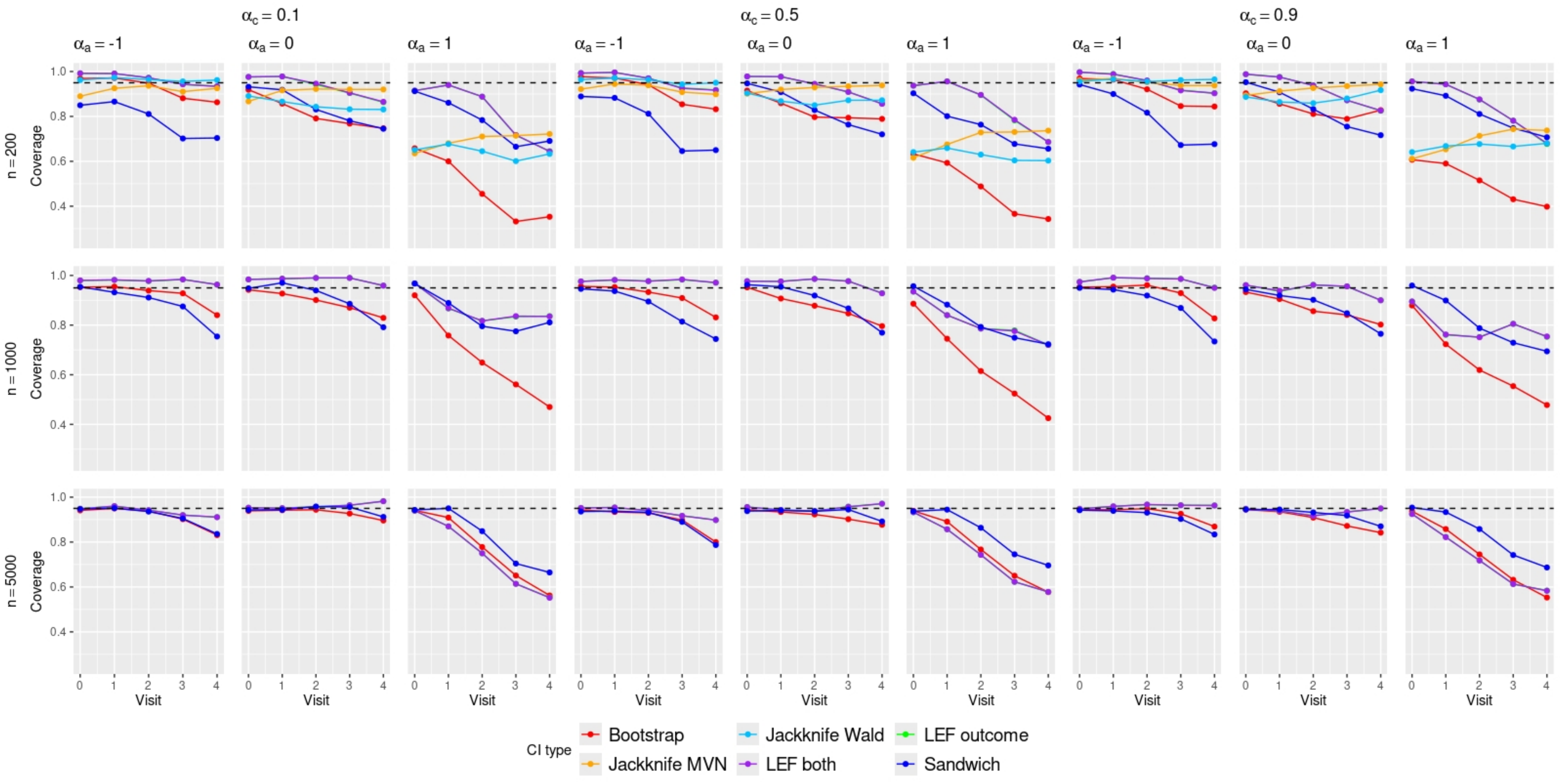

Figure 4: Bias-eliminated coverage of the CIs under **low event rates**. Bootstrap: CIs constructed by nonparametric bootstrap; LEF both: CIs constructed by applying Approach 2 of LEF bootstrap; LEF outcome: CIs constructed by applying Approach 1 of LEF bootstrap; Jackknife Wald: CIs constructed by applying Approach 1 of Jackknife resampling; Jackknife MVN: CIs constructed by applying Approach 2 of Jackknife resampling; Sandwich: CIs based on the sandwich variance estimator

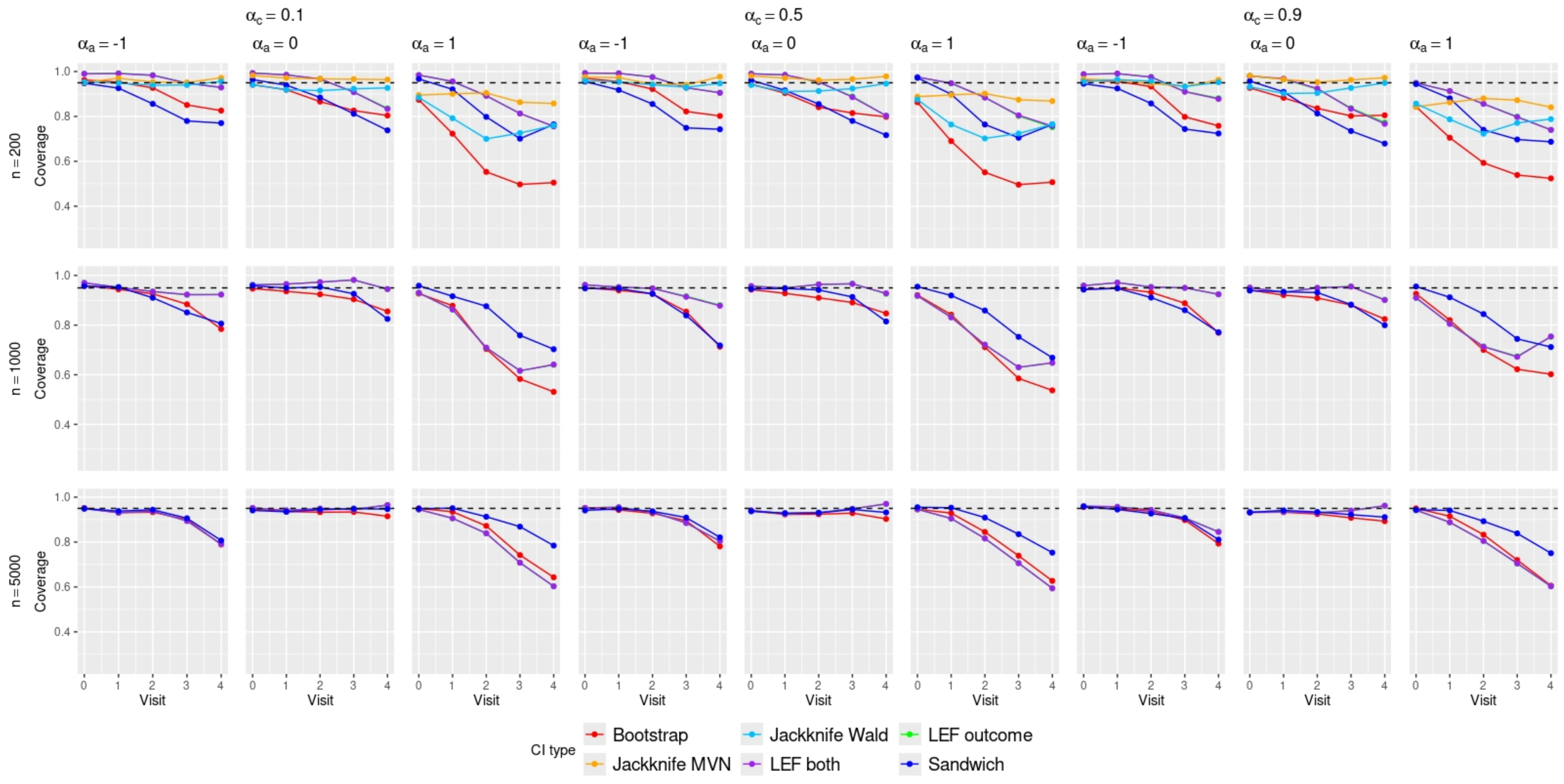

Figure 5: Bias-eliminated coverage of the CIs under **medium event rates**. Bootstrap: CIs constructed by nonparametric bootstrap; LEF both: CIs constructed by applying Approach 2 of LEF bootstrap; LEF outcome: CIs constructed by applying Approach 1 of LEF bootstrap; Jackknife Wald: CIs constructed by applying Approach 1 of Jackknife resampling; Jackknife MVN: CIs constructed by applying Approach 2 of Jackknife resampling; Sandwich: CIs based on the sandwich variance estimator

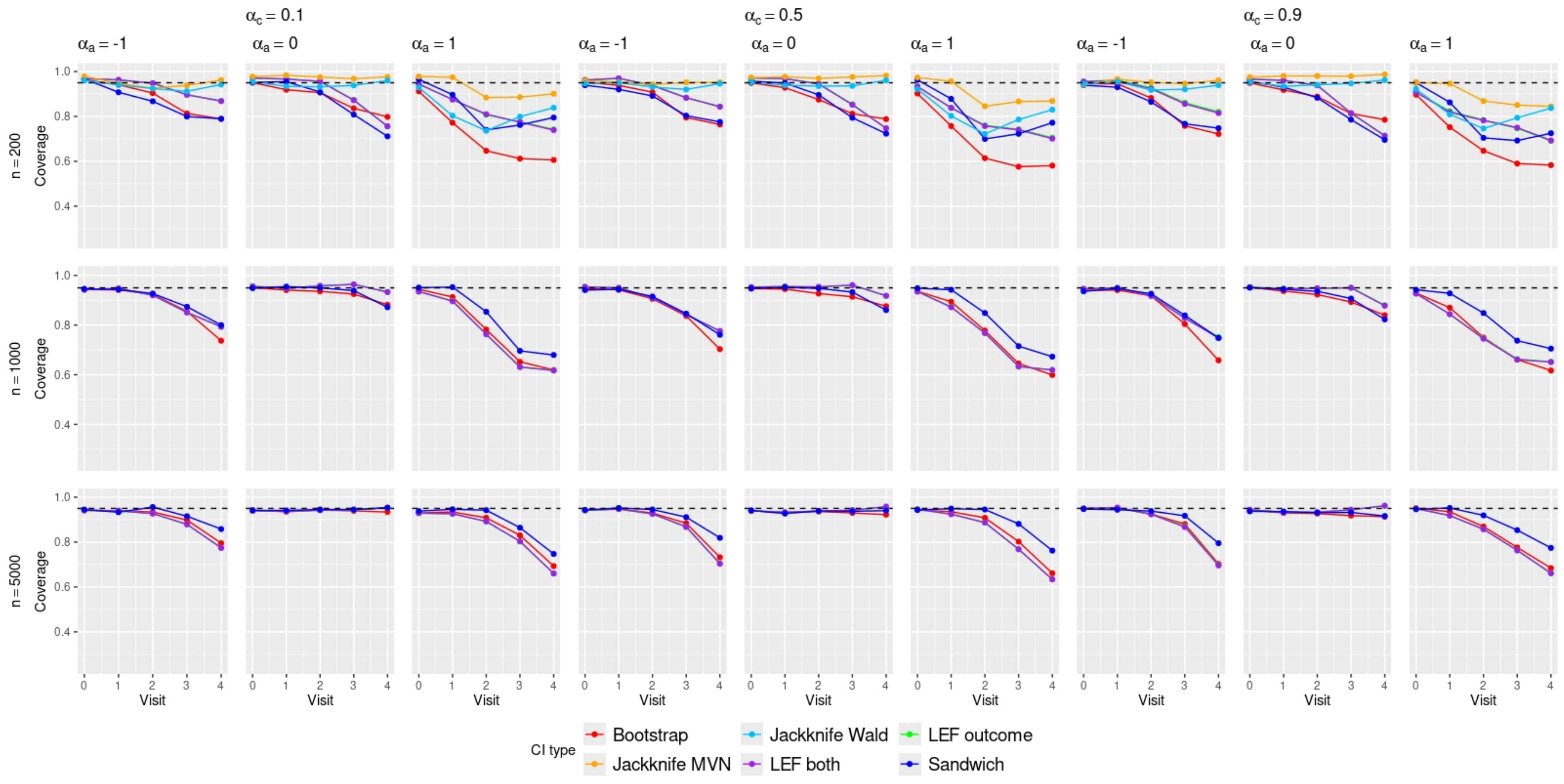

Figure 6: Bias-eliminated coverage of the CIs under **high event rates**. Bootstrap: CIs constructed by nonparametric bootstrap; LEF both: CIs constructed by applying Approach 2 of LEF bootstrap; LEF outcome: CIs constructed by applying Approach 1 of LEF bootstrap; Jackknife Wald: CIs constructed by applying Approach 1 of Jackknife resampling; Jackknife MVN: CIs constructed by applying Approach 2 of Jackknife resampling; Sandwich: CIs based on the sandwich variance estimator

5.4 ‘Pseudo-true’ marginal risk differences and resulting CI coverage

Following a reviewer’s suggestion, we also calculated the CI coverage rates using ‘pseudo-true’ marginal risk difference (MRD) implied by the misspecified marginal structural model (MSM) instead of the true values from the Kaplan-Meier (K-M) curve estimates to investigate the validity of the presented coverage results given the misspecified MSM. Recall that the discrete-time hazard at the baseline visit of the trials was modelled correctly in the MSM. Nonetheless, logistic models of the discrete-time hazards at later visits were not correctly specified due to non-collapsibility.

To obtain these ‘pseudo-true’ MRDs, we fitted the same weight estimation models and MSM to 200 large simulated data sets, each of 200,000 patients, and then averaged the 200 resulting MRD estimates. The absolute differences between the resulting ‘pseudo-true’ MRDs and the corresponding K-M curve estimates of the true MRDs were at most 0.001 in almost all simulation scenarios (i.e. scenarios not highlighted by colours in Table 10). A difference of 0.001 or less is negligible compared to the empirical standard deviation (SD) of the MRD estimates obtained from a data set of size $n = 5000$ or smaller (see Table 11 for the empirical SD of MRD estimates from 1000 simulated data sets with the sample size $n = 5000$ and also Figure 10). Consequently, coverages of confidence intervals would be expected to be almost identical whether we use the ‘pseudo-true’ values or the K-M curve estimates of the MRDs as the ‘true’ value of MRDs (and this also applies to the empirical biases of the point estimates of the MRDs).

Table 10: Differences between ‘pseudo-true’ MRDs calculated by averaging MRD estimates from 200 data sets with 200,000 patients (700 data sets for scenarios in blue, 1000 data sets for scenario in pink) versus the K-M curve estimates up to the third decimal place, by outcome event rate, trial visit, confounding strength α_c and treatment prevalence α_a.

Outcome	Visit		$\alpha_c = 0.1$			$\alpha_c = 0.5$			$\alpha_c = 0.9$	
event rate		$\alpha_a = -1$	$\alpha_a = 0$	$\alpha_a = 1$	$\alpha_a = -1$	$\alpha_a = 0$	$\alpha_a = 1$	$\alpha_a = -1$	$\alpha_a = 0$	$\alpha_a = 1$
Low	0	0.000	0.000	0.000	0.000	0.000	0.000	0.000	0.000	0.000
	1	0.000	0.000	0.000	0.000	0.000	0.000	0.000	0.001	0.000
	2	0.000	0.000	0.000	0.000	0.000	0.000	0.000	0.001	0.000
	3	0.000	0.000	0.000	0.000	0.000	-0.001	0.000	0.001	0.000
	4	0.000	0.000	0.000	0.000	0.000	-0.001	0.000	0.001	0.001
Medium	0	0.000	0.000	0.000	0.000	0.000	0.001	0.000	0.000	-0.001
	1	0.000	0.000	0.000	0.000	0.000	0.000	0.000	0.000	-0.001
	2	0.000	0.000	-0.001	0.001	0.000	0.001	0.000	-0.001	-0.001
	3	0.000	0.000	-0.001	0.001	0.000	0.001	0.000	-0.001	0.001
	4	0.000	0.000	-0.001	0.001	0.001	0.001	0.000	-0.001	0.001
High	0	0.000	0.000	-0.001	0.000	0.000	0.001	0.000	0.000	0.001
	1	0.000	0.001	0.000	0.000	0.000	0.000	0.000	0.000	0.000
	2	0.000	0.001	0.000	0.000	0.000	0.000	0.000	0.000	0.000
	3	0.000	0.001	0.001	-0.001	0.001	0.001	0.000	0.000	0.000
	4	0.001	0.001	0.001	-0.001	0.001	0.000	0.001	0.001	0.001

Table 11: Empirical SD of MRD estimates from 1000 simulated data sets with the sample size of $n = 5000$, by outcome event rate, trial visit, confounding strength α_c and treatment prevalence α_a.

Outcome event rate	Visit	$\alpha_c = 0.1$			$\alpha_c = 0.5$			$\alpha_c = 0.9$		
		$\alpha_a = -1$	$\alpha_a = 0$	$\alpha_a = 1$	$\alpha_a = -1$	$\alpha_a = 0$	$\alpha_a = 1$	$\alpha_a = -1$	$\alpha_a = 0$	$\alpha_a = 1$
Low	0	0.002	0.002	0.003	0.002	0.003	0.004	0.002	0.003	0.005
	1	0.004	0.005	0.008	0.004	0.006	0.010	0.005	0.007	0.013
	2	0.007	0.008	0.018	0.008	0.010	0.021	0.010	0.013	0.029
	3	0.014	0.013	0.036	0.016	0.015	0.041	0.018	0.024	0.052
	4	0.030	0.021	0.060	0.047	0.026	0.066	0.052	0.039	0.076
Medium	0	0.003	0.004	0.005	0.003	0.004	0.005	0.003	0.005	0.006
	1	0.006	0.007	0.012	0.006	0.008	0.013	0.007	0.010	0.017
	2	0.011	0.012	0.024	0.012	0.014	0.028	0.015	0.018	0.037
	3	0.020	0.018	0.048	0.024	0.021	0.054	0.029	0.030	0.068
	4	0.044	0.029	0.078	0.056	0.035	0.094	0.076	0.047	0.103
High	0	0.004	0.005	0.007	0.004	0.006	0.007	0.005	0.006	0.008
	1	0.009	0.010	0.015	0.009	0.011	0.017	0.010	0.013	0.021
	2	0.015	0.015	0.030	0.016	0.017	0.033	0.020	0.022	0.044
	3	0.028	0.023	0.058	0.032	0.026	0.064	0.039	0.036	0.079
	4	0.059	0.035	0.103	0.080	0.040	0.112	0.106	0.056	0.119

In a few simulation scenarios, the absolute difference between the 'pseudo-true' MRD estimated from 200 large data sets and the K-M curve estimate of the true MRD was greater than 0.001. These scenarios (highlighted by colours in Table 10) were: 1) low event rate, medium confounding strength, high treatment prevalence; 2) low event rate, strong confounding strength, high treatment prevalence; 3) high event rate, strong confounding strength, high treatment prevalence; and 4) high event rate, strong confounding strength, low treatment prevalence. Monte Carlo error was the reason for the greater difference in these scenarios. When we increased the number of large simulated data sets from 200 to 700 or 1000, we obtained absolute differences of 0.001 or less.

We re-estimated the coverage rates of the CIs methods using the 'pseudo-true' MRD values. The results are presented in Figures 7, 8, 9 below. By comparing these with the coverage results based on true MRD values calculated by the K-M curve method (Figure 1 of the main text and Figure 2 and 3 earlier), we conclude that the differences in estimated coverage rates are very small and do not affect our conclusions about the coverage performance of the CI methods in Section 5.2 of the main text.

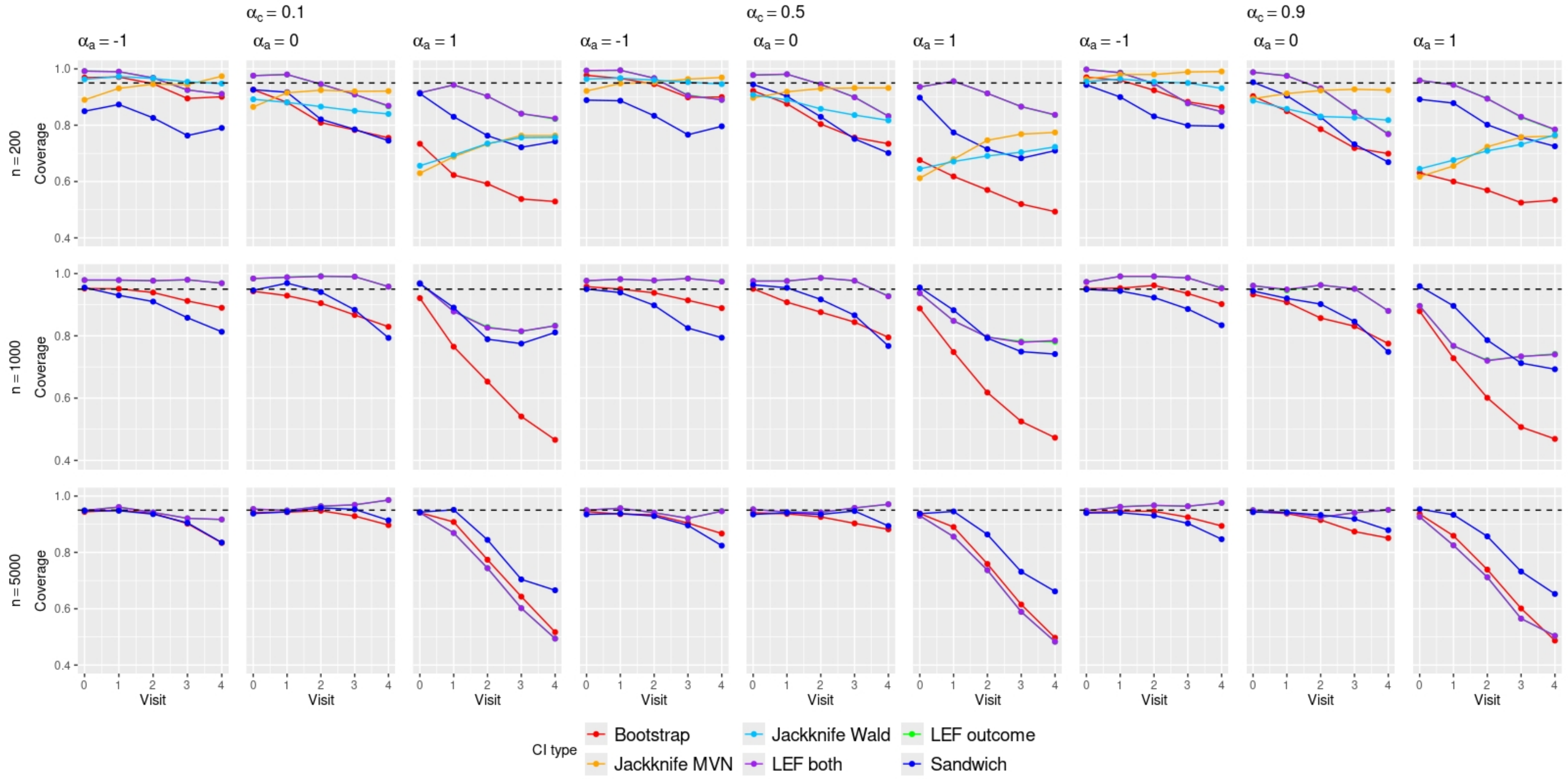

Figure 7: Empirical coverage of the CIs *using the 'pseudo-true' MRDs induced by the misspecified MSM* in the scenarios with **low event rates**. Bootstrap: CIs constructed by nonparametric bootstrap. LEF both: CIs constructed by applying Approach 2 of LEF bootstrap. LEF outcome: CIs constructed by applying Approach 1 of LEF bootstrap. Jackknife Wald: CIs constructed by applying Approach 1 of jackknife resampling; jackknife MVN: CIs constructed by applying Approach 2 of jackknife resampling; Sandwich: CIs based on the sandwich variance estimator. Note that the results for applying Approaches 1 and 2 of LEF bootstrap were very similar so that the purple and green lines overlapped.

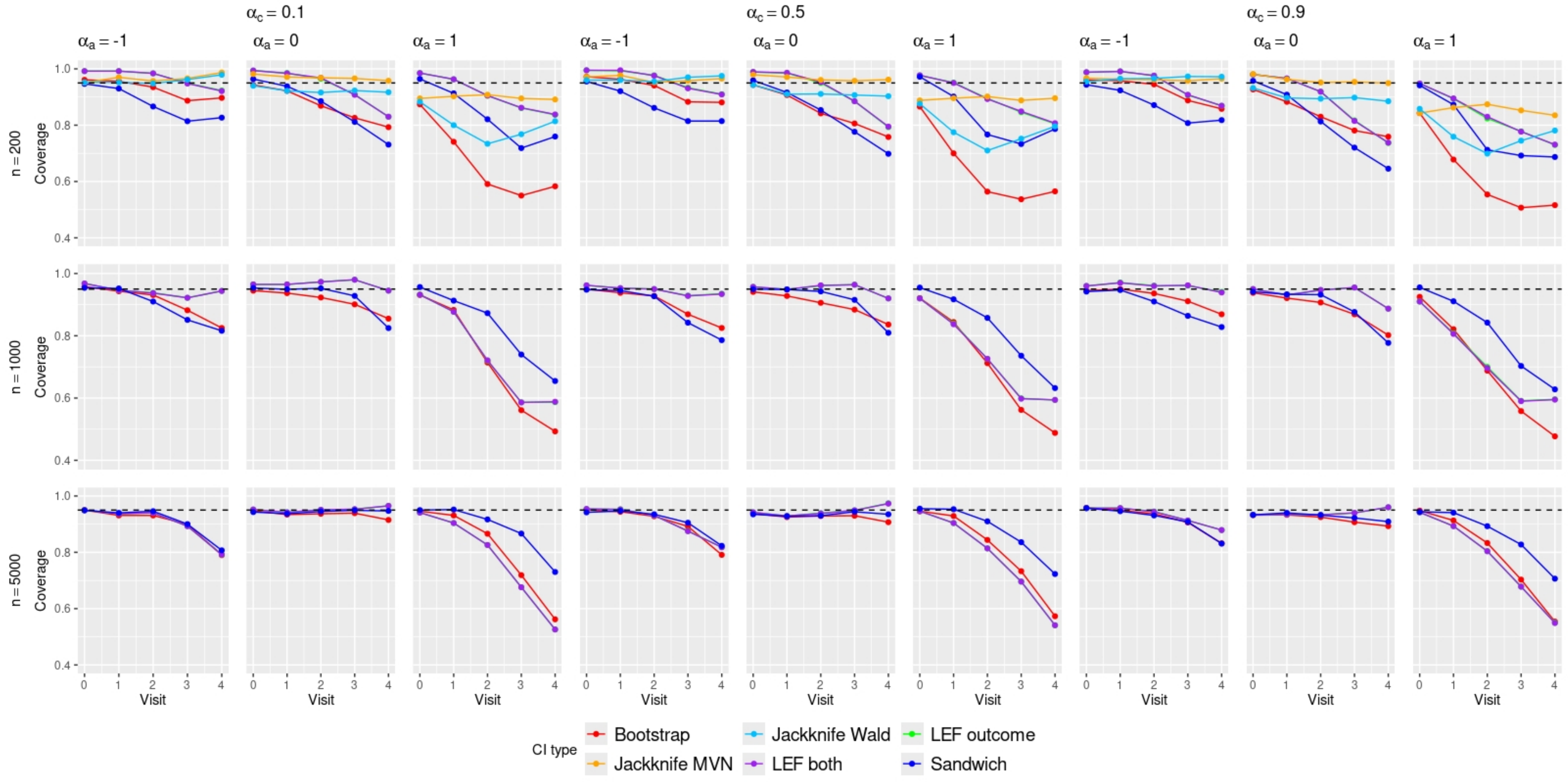

Figure 8: Empirical coverage of the CIs *using the 'pseudo-true' MRDs induced by the misspecified MSM* in the scenarios with **medium event rates**. Bootstrap: CIs constructed by nonparametric bootstrap. LEF both: CIs constructed by applying Approach 2 of LEF bootstrap. LEF outcome: CIs constructed by applying Approach 1 of LEF bootstrap. Jackknife Wald: CIs constructed by applying Approach 1 of jackknife resampling; jackknife MVN: CIs constructed by applying Approach 2 of jackknife resampling; Sandwich: CIs based on the sandwich variance estimator. Note that the results for applying Approaches 1 and 2 of LEF bootstrap were very similar so that the purple and green lines overlapped.

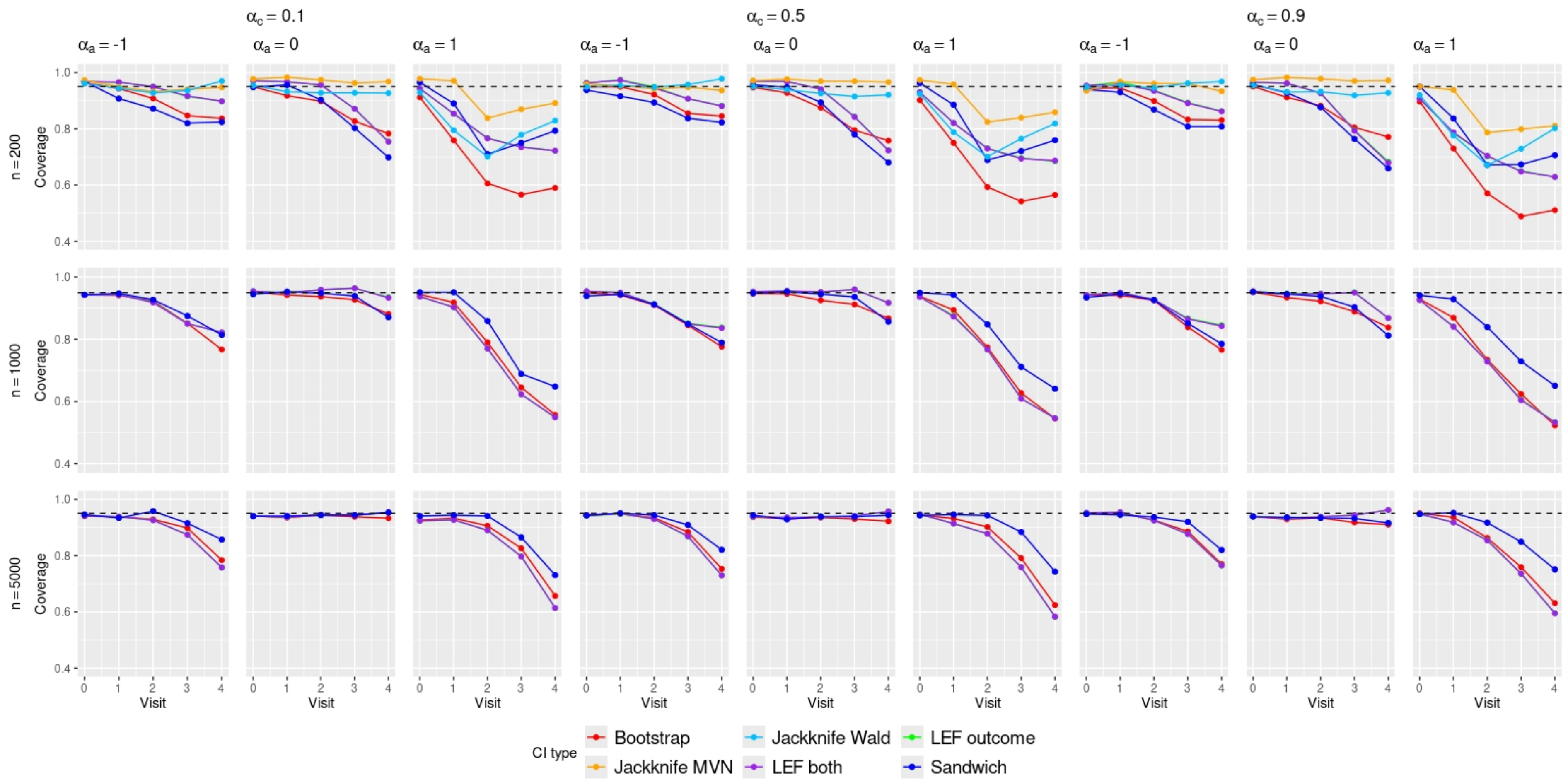

Figure 9: Empirical coverage of the CIs *using the 'pseudo-true' MRDs induced by the misspecified MSM* in the scenarios with **high event rates**. Bootstrap: CIs constructed by nonparametric bootstrap. LEF both: CIs constructed by applying Approach 2 of LEF bootstrap. LEF outcome: CIs constructed by applying Approach 1 of LEF bootstrap. Jackknife Wald: CIs constructed by applying Approach 1 of jackknife resampling; jackknife MVN: CIs constructed by applying Approach 2 of jackknife resampling; Sandwich: CIs based on the sandwich variance estimator. Note that the results for applying Approaches 1 and 2 of LEF bootstrap were very similar so that the purple and green lines overlapped.

5.5 Empirical standard deviation and MSE of the MRD estimates, and empirical distribution of IPTWs

For all scenarios examined, the empirical standard deviations (Figure 10) were always higher at later visits than at earlier visits. There are a few possible reasons for this: 1) due to the non-collapsibility of logistic models, we were not able to specify an exactly correct MSM of the potential outcomes parameterised by the discrete-time hazard. Instead, we used a rich model stratified by trial visits, which led to data sparsity and larger variability in MRD estimates at later visits. 2) Only earlier trials had data at longer follow-up trial visits, so the information was scarce by design. 3) As mentioned previously, we rely on IPW to estimate the MRD. The limited amount of observations at later visits paired with the increased imbalance between the treatment groups likely resulted in the estimated weights for later trials visits being larger and more variable. Consequently, the larger magnitude and variability of the weights at later visits could contribute to the unstable estimation of the MRD.

The patterns of empirical standard deviations largely followed those of empirical biases across treatment prevalence and confounding strength scenarios. That is, the scenarios with larger biases also exhibited larger standard deviations. Overall, the impact of treatment prevalence on empirical standard deviations was more prominent than that of confounding strength, possibly due to the small range we specified for the coefficients of the time-varying confounder in the treatment and outcome processes. Interestingly, the empirical standard deviations increased when event rates were higher, when fixing the sample size, treatment prevalence and confounding strength. This phenomenon might be explained by that more patients from the untreated group were depleted from the at-risk sets due to more event occurrences, which resulted in larger treatment group imbalances at later visits. As expected, larger sample sizes mitigated this variability by providing more information. Results for the root-MSE were consistent with the results for bias and empirical standard deviations and can be found in Figure 11.

Table 12 presents summary statistics of the estimated IPTWs stratified by assigned treatments from a large data set with $n = 50,000$ for each simulation scenario.

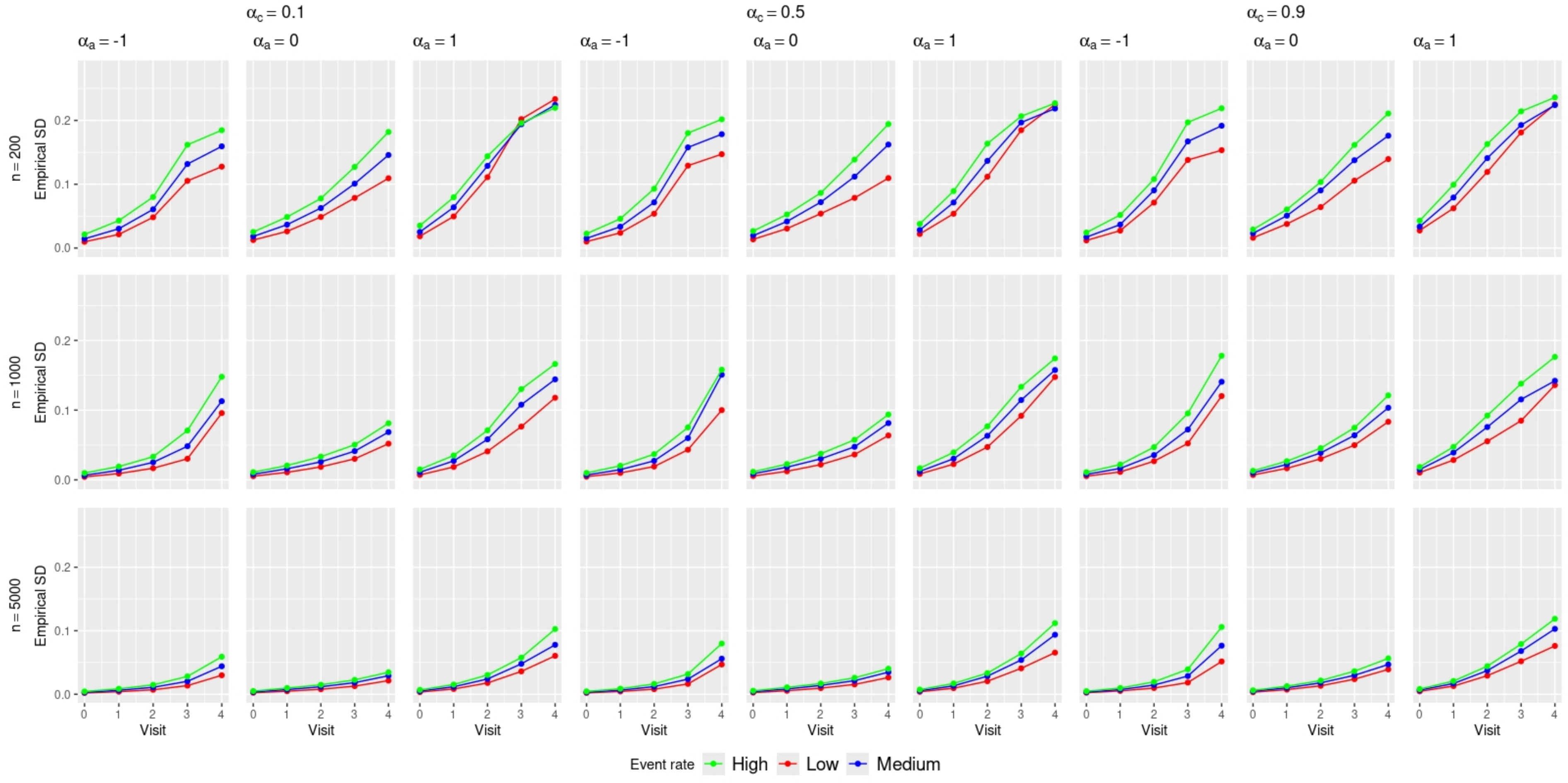

Figure 10: Empirical standard deviation of the MRD estimates in various simulation scenarios.

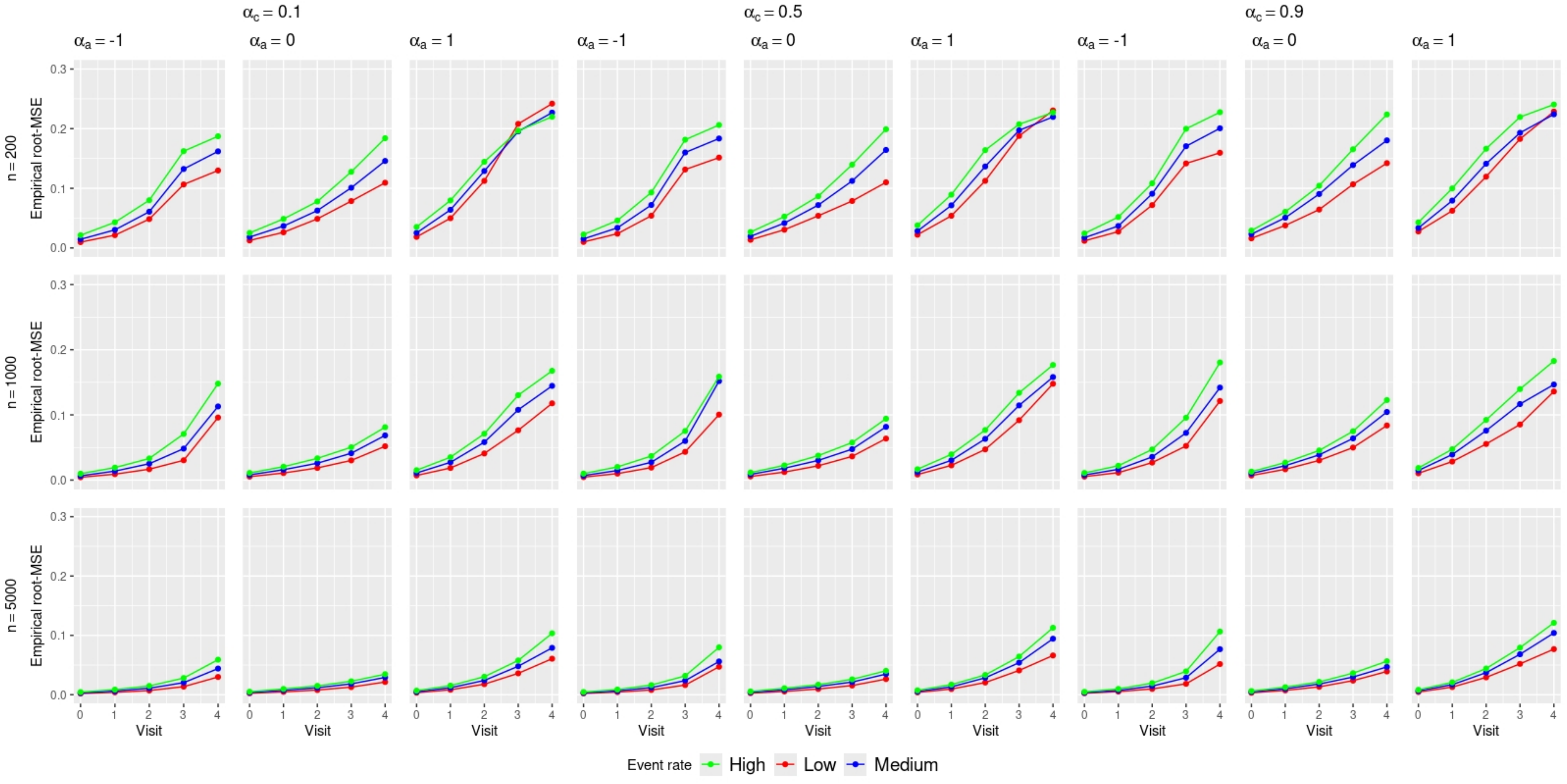

Figure 11: Root-mean-squared error (MSE) of the MRD estimates in various simulation scenarios.

Table 12: Summary statistics of the estimated IPTWs for one simulated data set from each scenario with $n = 50,000$, stratified by assigned treatments.

Outcome event rate	Confounding strength	Treatment prevalence	Assigned treatment	Minimum	1st quantile	Mean	Median	3rd quantile	Maximum
Low	0.1	-1	0	0.53	0.97	1.00	1.00	1.00	2.31
			1	0.20	1.00	1.00	1.00	1.00	3.05
		0	0	0.28	0.98	0.99	1.00	1.00	4.35
			1	0.30	1.00	1.00	1.00	1.00	3.09
		1	0	0.18	1.00	0.99	1.00	1.00	4.42
			1	0.58	0.97	1.00	1.00	1.01	2.33
	0.5	-1	0	0.40	0.93	1.00	1.00	1.00	5.16
			1	0.10	1.00	1.00	1.00	1.00	7.57
		0	0	0.20	0.94	0.99	1.00	1.00	6.24
			1	0.19	0.97	1.00	1.00	1.00	11.93
		1	0	0.09	1.00	0.99	1.00	1.00	8.70
			1	0.36	0.91	1.00	1.00	1.00	6.78
	0.9	-1	0	0.31	0.86	0.99	1.00	1.00	15.01
			1	0.05	1.00	1.00	1.00	1.00	34.08
		0	0	0.14	0.85	0.99	1.00	1.00	18.88
			1	0.09	0.91	1.00	1.00	1.00	46.05
		1	0	0.05	1.00	1.00	1.00	1.00	25.61
			1	0.26	0.83	1.00	1.00	1.00	13.35
Medium	0.1	-1	0	0.53	0.97	0.99	1.00	1.00	2.26
			1	0.17	1.00	1.00	1.00	1.00	3.23
		0	0	0.28	0.98	0.99	1.00	1.00	3.82
			1	0.35	1.00	1.00	1.00	1.00	3.87
		1	0	0.23	1.00	0.99	1.00	1.00	3.45
			1	0.54	0.97	1.00	1.00	1.01	2.80
	0.5	-1	0	0.44	0.93	0.99	1.00	1.00	4.98
			1	0.09	1.00	1.00	1.00	1.00	9.53
		0	0	0.21	0.94	0.99	1.00	1.00	9.06
			1	0.18	0.98	1.00	1.00	1.00	11.23
		1	0	0.08	1.00	0.99	1.00	1.00	9.84
			1	0.38	0.91	1.00	1.00	1.00	6.10
	0.9	-1	0	0.30	0.86	0.99	1.00	1.00	19.87
			1	0.05	1.00	1.00	1.00	1.00	29.49
		0	0	0.11	0.85	0.99	1.00	1.00	25.19
			1	0.09	0.92	1.00	1.00	1.00	37.21
		1	0	0.03	1.00	0.99	1.00	1.00	22.47
			1	0.26	0.83	1.00	1.00	1.00	19.14
High	0.1	-1	0	0.53	0.97	0.99	1.00	1.00	2.23
			1	0.22	1.00	1.00	1.00	1.00	3.63
		0	0	0.30	0.98	0.99	1.00	1.00	3.00
			1	0.33	1.00	1.00	1.00	1.00	4.63
		1	0	0.25	1.00	0.99	1.00	1.00	2.35
			1	0.56	0.97	1.00	1.00	1.01	3.36
	0.5	-1	0	0.40	0.93	0.99	1.00	1.00	6.68
			1	0.12	1.00	1.00	1.00	1.00	8.92
		0	0	0.20	0.94	0.99	1.00	1.00	6.53
			1	0.17	0.99	1.00	1.00	1.00	10.53
		1	0	0.12	1.00	0.99	1.00	1.00	7.45
			1	0.37	0.92	1.00	1.00	1.00	8.46
	0.9	-1	0	0.30	0.86	0.99	1.00	1.00	12.33
			1	0.05	1.00	1.00	1.00	1.00	41.07
		0	0	0.11	0.86	0.99	1.00	1.00	22.12
			1	0.11	0.96	1.00	1.00	1.00	23.73
		1	0	0.04	1.00	0.99	1.00	1.00	17.42
			1	0.25	0.84	1.01	1.00	1.00	41.92

5.6 Ratio of estimated standard errors to the empirical standard deviation of the MRD estimator

Figure 12 presents the SE ratio results in the scenarios with low event rate and moderate/large sample sizes. Figures 13–18 present the SE ratio results in the medium and high event rate scenarios.

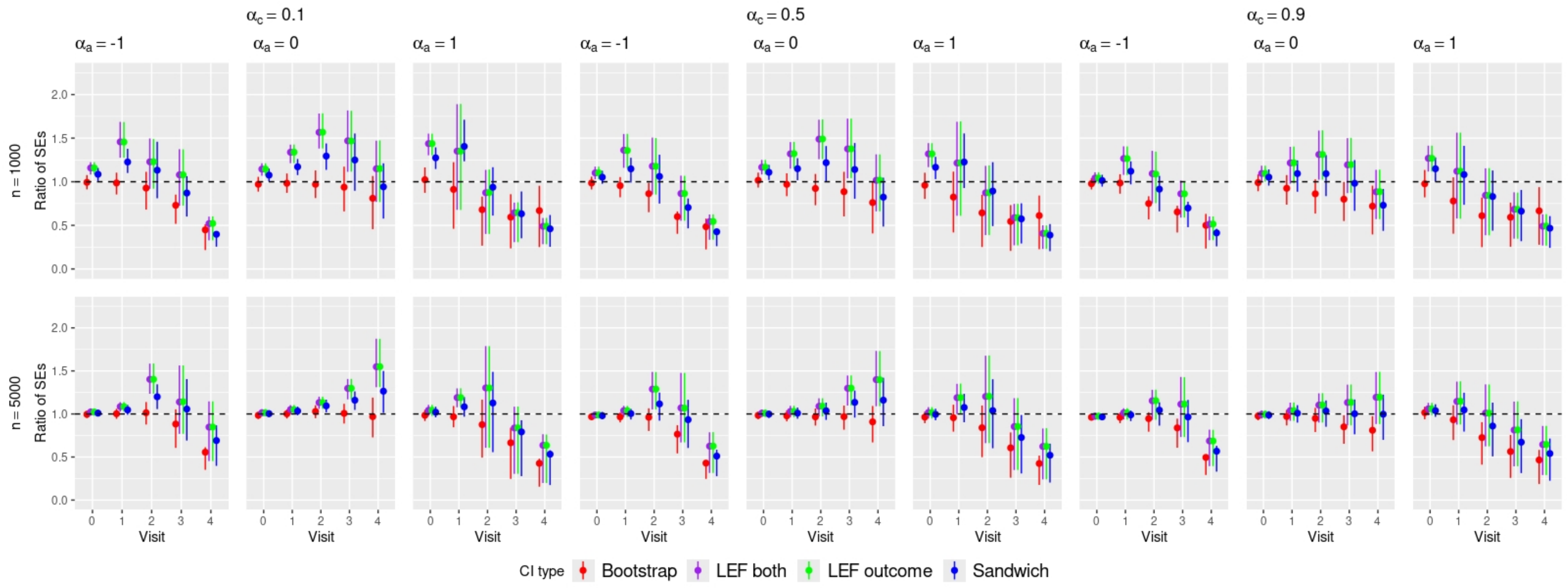

Figure 12: Ratio of the estimated standard error to empirical standard deviation of the MRD estimator (SE ratio) in **low event rate and medium/large sample size scenarios** ($n = 1000, 5000$). The dots represent the averages of the ratio, with the bottom and top of the bar being the 1st and 3rd quartile of this ratio respectively. Bootstrap: CIs constructed by nonparametric bootstrap; LEF both: CIs constructed by applying Approach 2 of LEF bootstrap; LEF outcome: CIs constructed by applying Approach 1 of LEF bootstrap; Jackknife Wald: CIs constructed by applying Approach 1 of jackknife resampling; Sandwich: CIs based on the sandwich variance estimator.

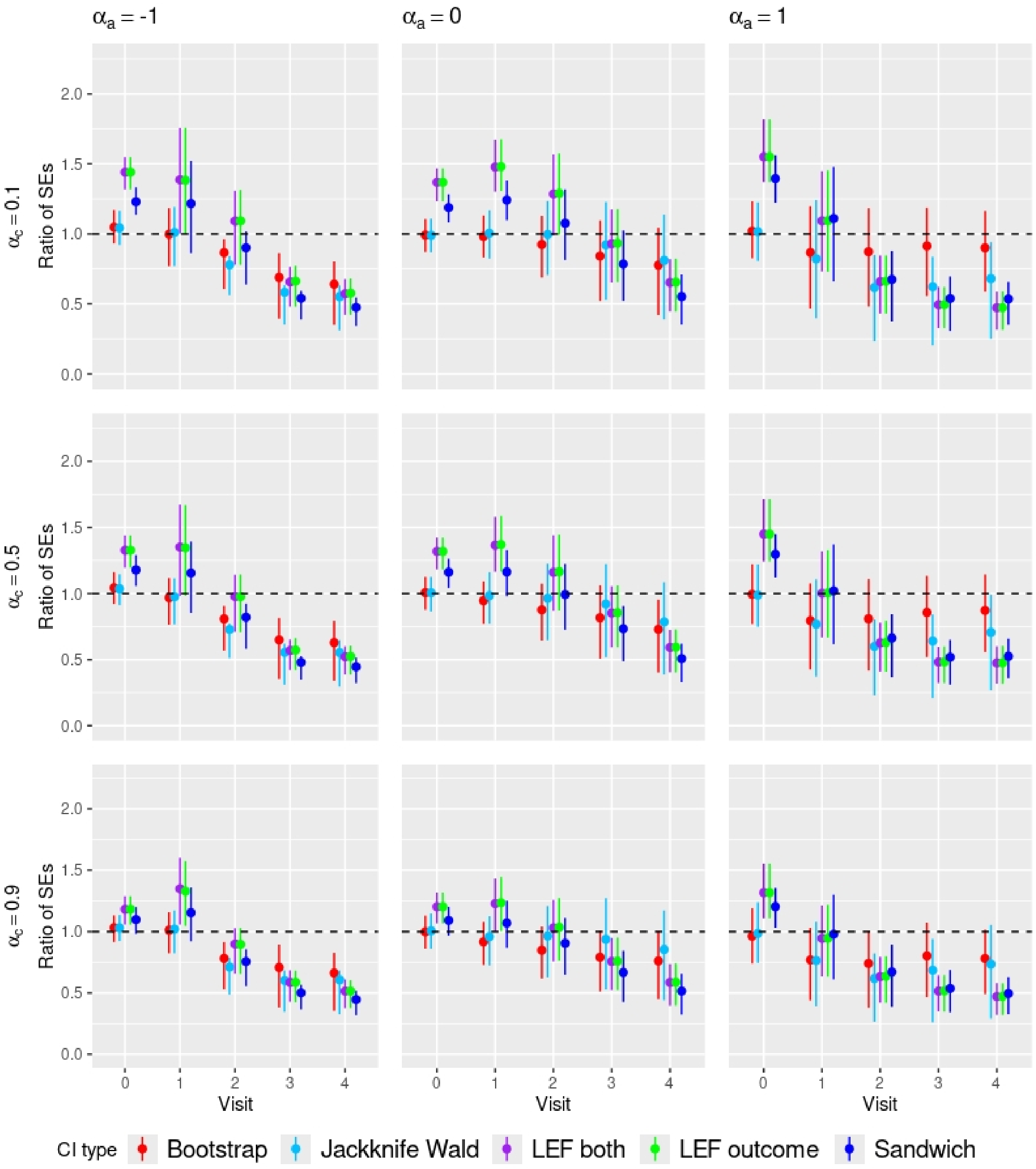

Figure 13: Ratio of the estimated standard error to empirical standard deviation of the MRD estimator (SE ratio) in **medium event rate and small sample size scenarios**. The dots represent the averages of the ratio, with the bottom and top of the bar being the 1st and 3rd quartile of this ratio respectively. Bootstrap: CIs constructed by nonparametric bootstrap; LEF both: CIs constructed by applying Approach 2 of LEF bootstrap; LEF outcome: CIs constructed by applying Approach 1 of LEF bootstrap; Jackknife Wald: CIs constructed by applying Approach 1 of jackknife resampling; Sandwich: CIs based on the sandwich variance estimator.

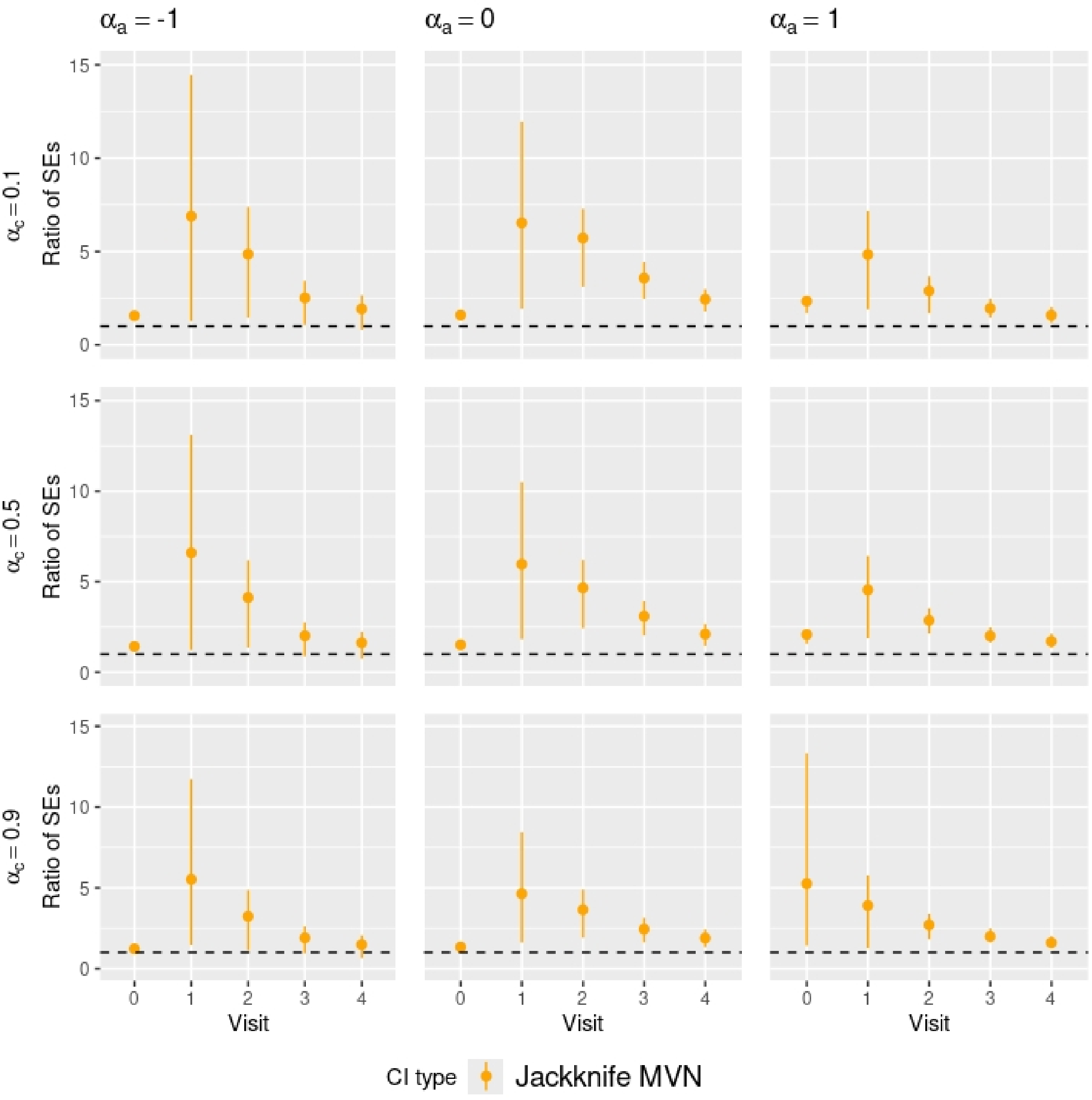

Figure 14: Ratio of the estimated standard error to empirical standard deviation of the MRD estimator (SE ratio) in **medium event rate and small sample size scenarios**. The dots represent the averages of the ratio, with the bottom and top of the bar being the 1st and 3rd quartile of this ratio respectively. Jackknife MVN: CIs constructed by applying Approach 2 of jackknife resampling.

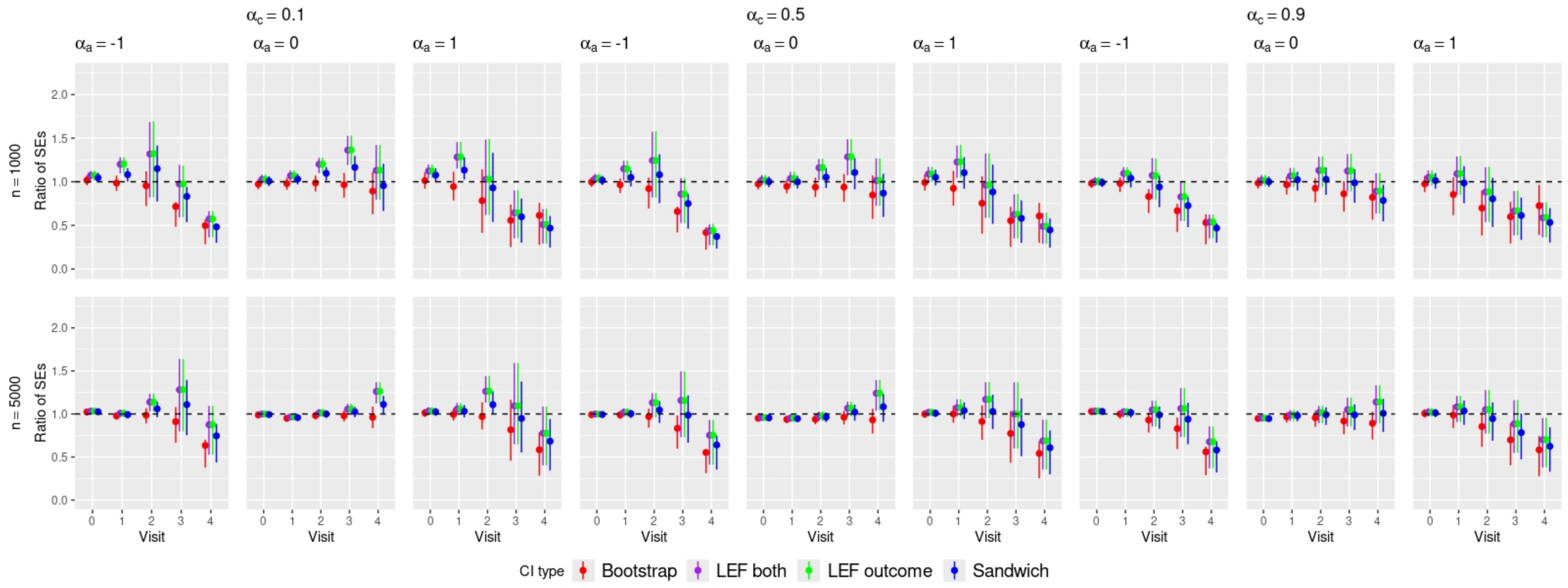

Figure 15: Ratio of the estimated standard error to empirical standard deviation of the MRD estimator (SE ratio) in **medium event rate and medium/large sample size scenarios** ($n = 1000, 5000$). The dots represent the averages of the ratio, with the bottom and top of the bar being the 1st and 3rd quartile of this ratio respectively. Bootstrap: CIs constructed by nonparametric bootstrap; LEF both: CIs constructed by applying Approach 2 of LEF bootstrap; LEF outcome: CIs constructed by applying Approach 1 of LEF bootstrap; Jackknife Wald: CIs constructed by applying Approach 1 of jackknife resampling; Sandwich: CIs based on the sandwich variance estimator.

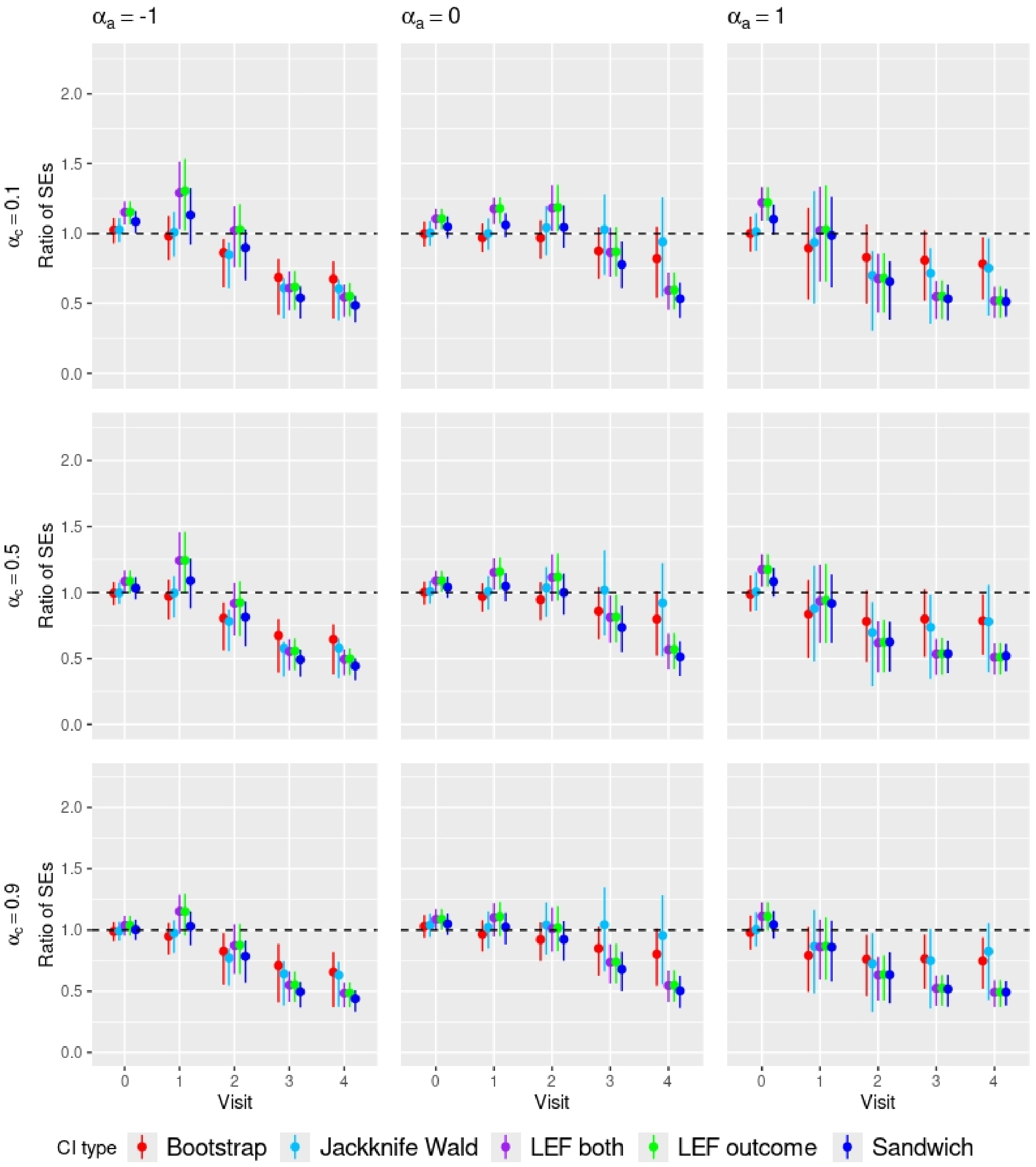

Figure 16: Ratio of the estimated standard error to empirical standard deviation of the MRD estimator (SE ratio) in **high event rate and small sample size scenarios**. The dots represent the averages of the ratio, with the bottom and top of the bar being the 1st and 3rd quartile of this ratio respectively. Bootstrap: CIs constructed by nonparametric bootstrap; LEF both: CIs constructed by applying Approach 2 of LEF bootstrap; LEF outcome: CIs constructed by applying Approach 1 of LEF bootstrap; Jackknife Wald: CIs constructed by applying Approach 1 of jackknife resampling; Sandwich: CIs based on the sandwich variance estimator.

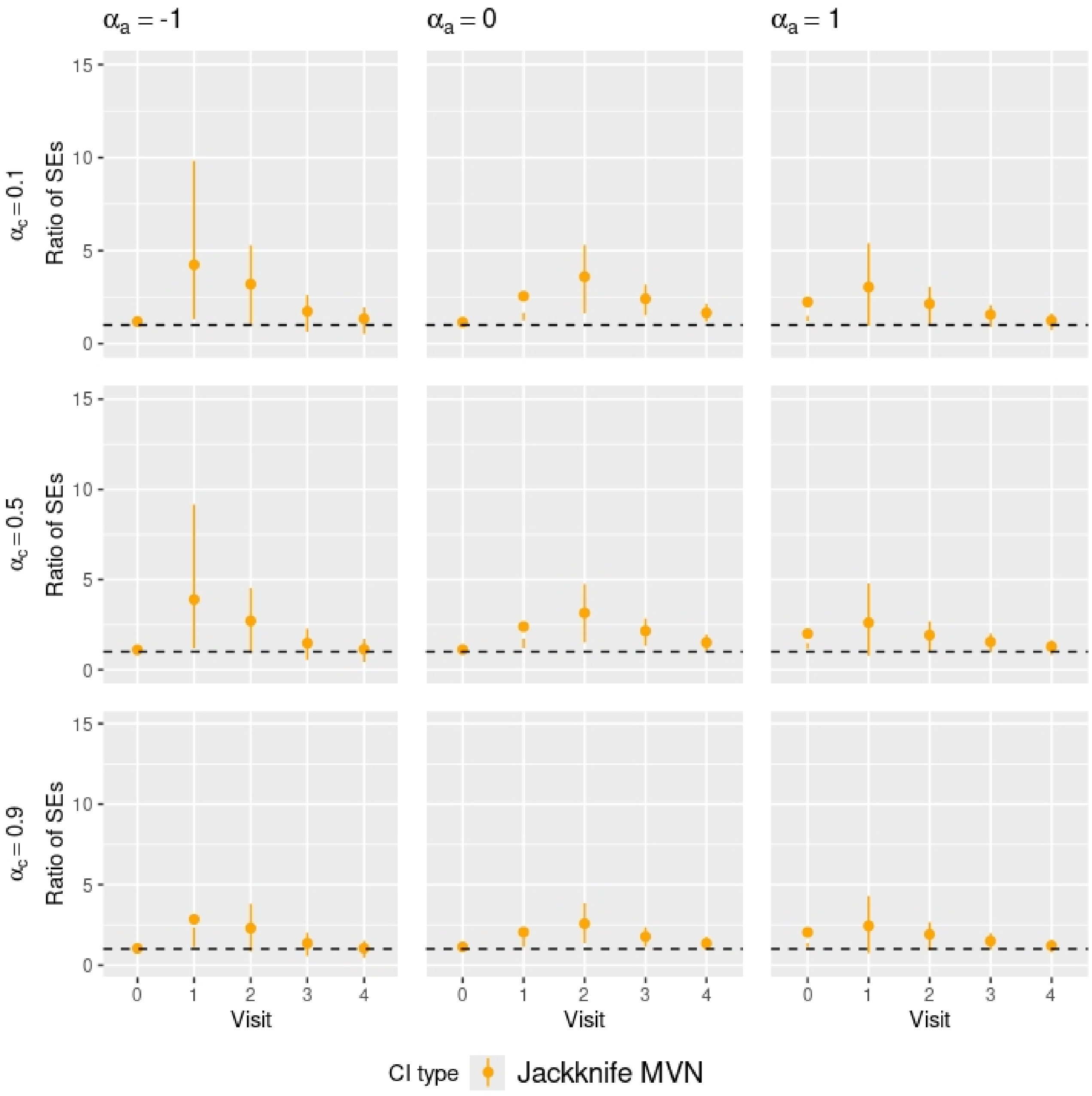

Figure 17: Ratio of the estimated standard error to empirical standard deviation of the MRD estimator (SE ratio) in **high event rate and small sample size scenarios**. The dots represent the averages of the ratio, with the bottom and top of the bar being the 1st and 3rd quartile of this ratio respectively. Jackknife MVN: CIs constructed by applying Approach 2 of jackknife resampling.

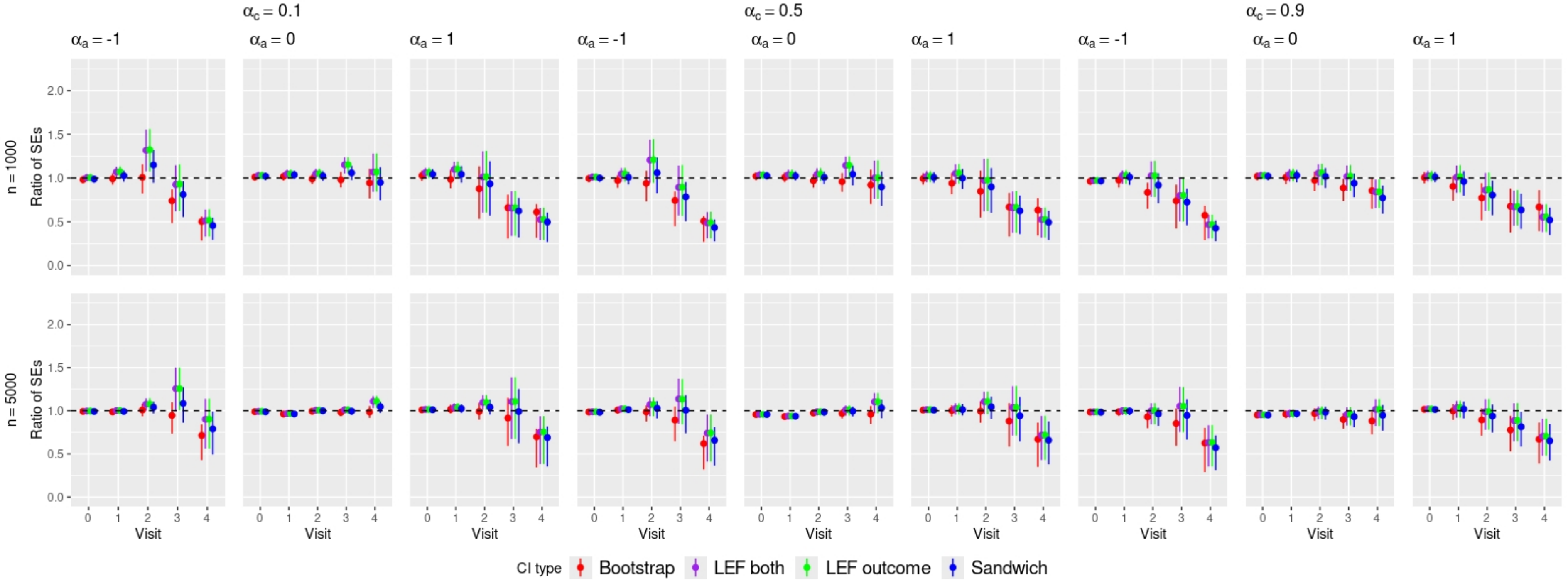

Figure 18: Ratio of the estimated standard error to empirical standard deviation of the MRD estimator (SE ratio) in **high event rate and medium/large sample size scenarios** ($n = 1000, 5000$). The dots represent the averages of the ratio, with the bottom and top of the bar being the 1st and 3rd quartile of this ratio respectively. Bootstrap: CIs constructed by nonparametric bootstrap; LEF both: CIs constructed by applying Approach 2 of LEF bootstrap; LEF outcome: CIs constructed by applying Approach 1 of LEF bootstrap; Jackknife Wald: CIs constructed by applying Approach 1 of jackknife resampling; Sandwich: CIs based on the sandwich variance estimator.

5.7 Monte Carlo standard error of the CI coverage

Finally, to assess whether lower-than-nominal coverage in some simulation scenarios may be due to uncertainty induced by Monte Carlo simulation error, we calculated and illustrated the standard errors of empirical coverage rates in Figures 19–21. The Monte Carlo standard errors of coverage rates across all simulation scenarios were under 1.75%, especially for small sample sizes where we did not observe patterns in Monte Carlo errors between the CI methods. According to the Monte Carlo standard error formula for CI coverage, if the expected coverage is 95% and we want to acheive a standard error of 1.75% or less across the simulations, then $n_{sim} = \frac{95(100-95)}{1.75^2} \approx 155$ simulations are required [5]. As a result, 1000 simulations were sufficient to theoretically achieve nominal coverage with a Monte Carlo error of at most 1.75% across the simulations. We also observed lower Monte Carlo errors at earlier visits in large sample size ($n = 5000$) scenarios. As the outcome event rate increased, the Monte Carlo errors converged to similar values for all CI methods as well.

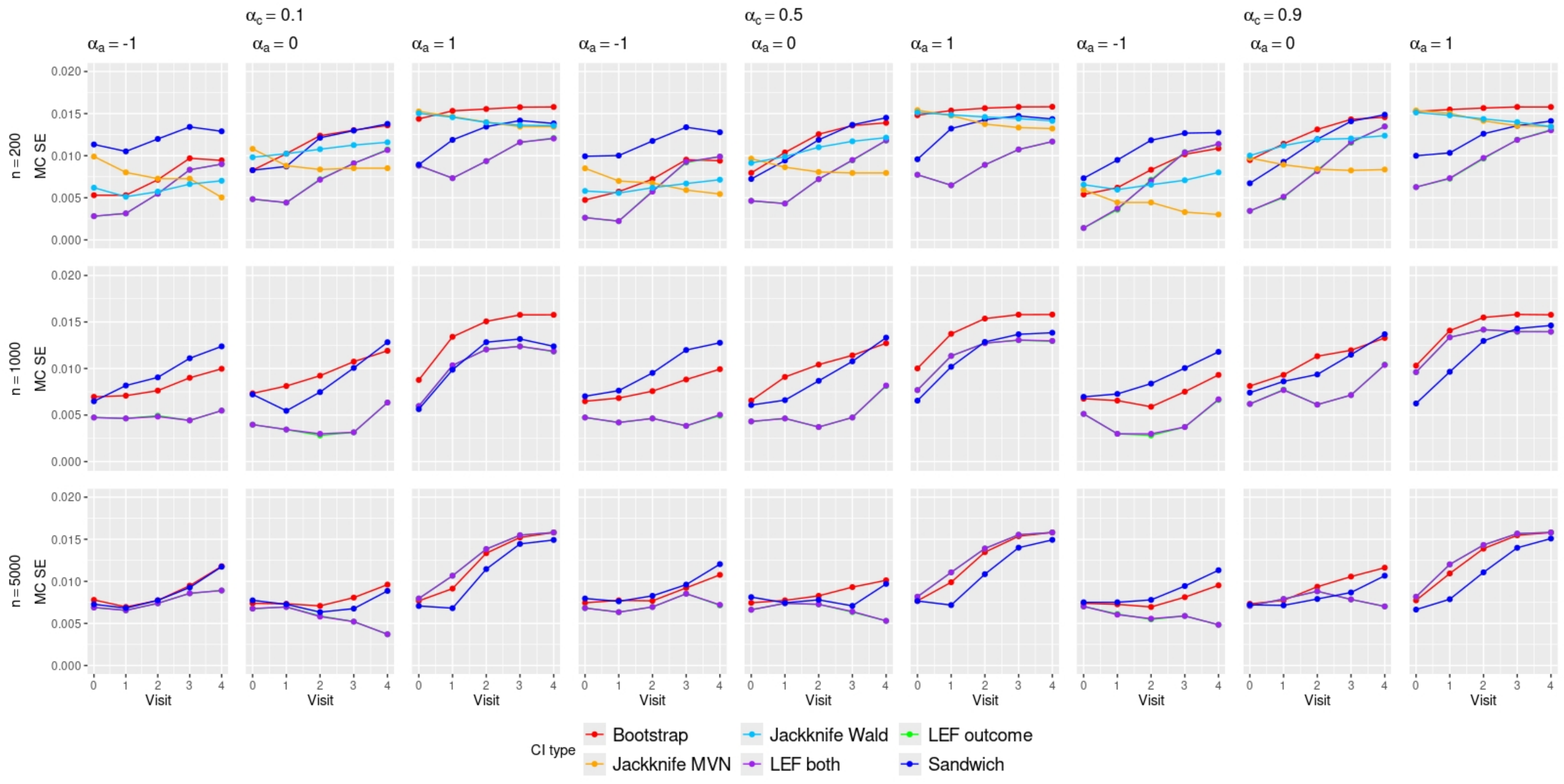

Figure 19: Monte Carlo standard error of the CI coverage under **low event rates**. Bootstrap: CIs constructed by nonparametric bootstrap; LEF both: CIs constructed by applying Approach 2 of LEF bootstrap; LEF outcome: CIs constructed by applying Approach 1 of LEF bootstrap; Jackknife Wald: CIs constructed by applying Approach 1 of Jackknife resampling; Jackknife MVN: CIs constructed by applying Approach 2 of Jackknife resampling; Sandwich: CIs based on the sandwich variance estimator

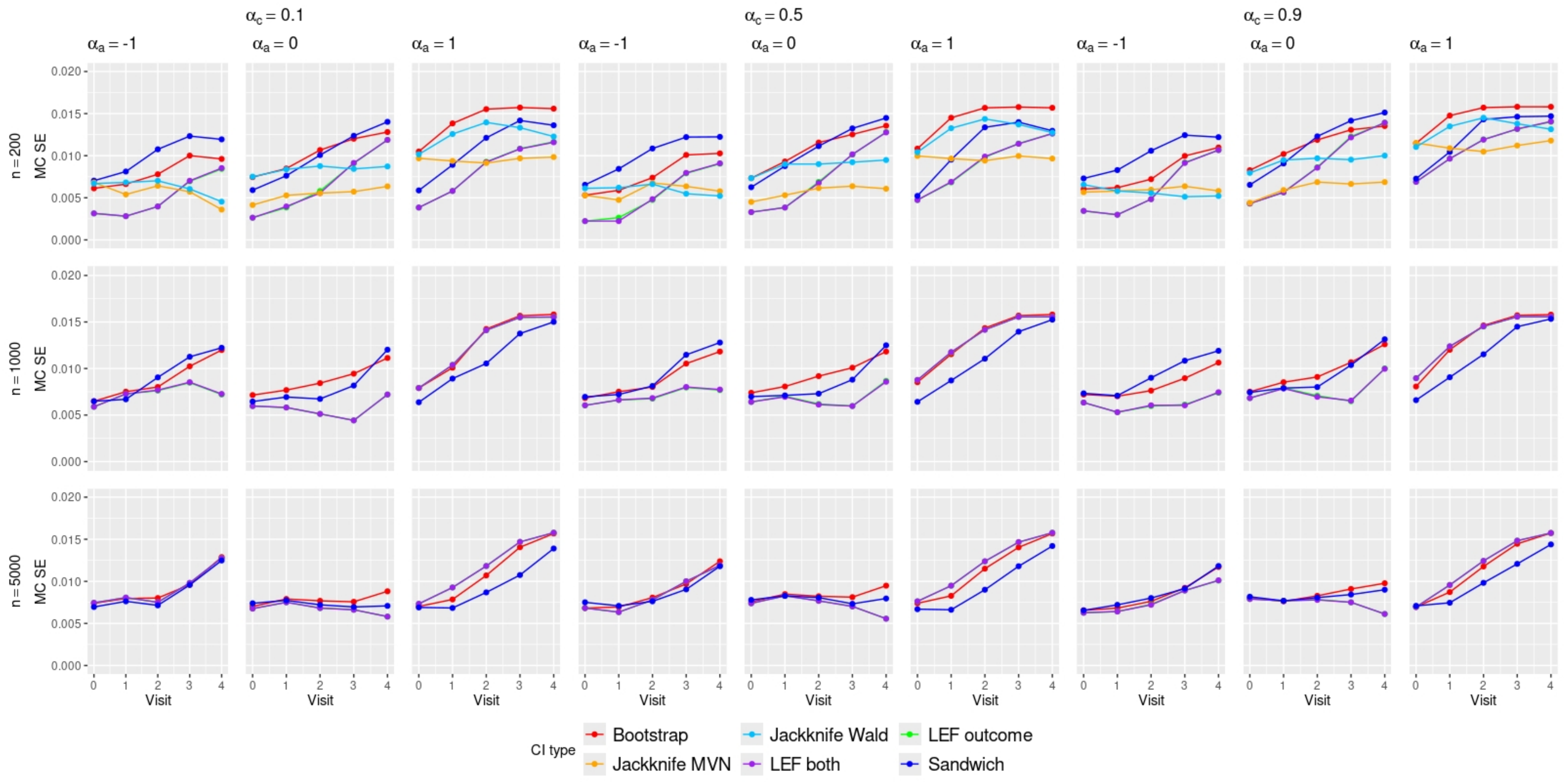

Figure 20: Monte Carlo standard error of the CI coverage under **medium event rates**. Bootstrap: CIs constructed by nonparametric bootstrap; LEF both: CIs constructed by applying Approach 2 of LEF bootstrap; LEF outcome: CIs constructed by applying Approach 1 of LEF bootstrap; Jackknife Wald: CIs constructed by applying Approach 1 of Jackknife resampling; Jackknife MVN: CIs constructed by applying Approach 2 of Jackknife resampling; Sandwich: CIs based on the sandwich variance estimator

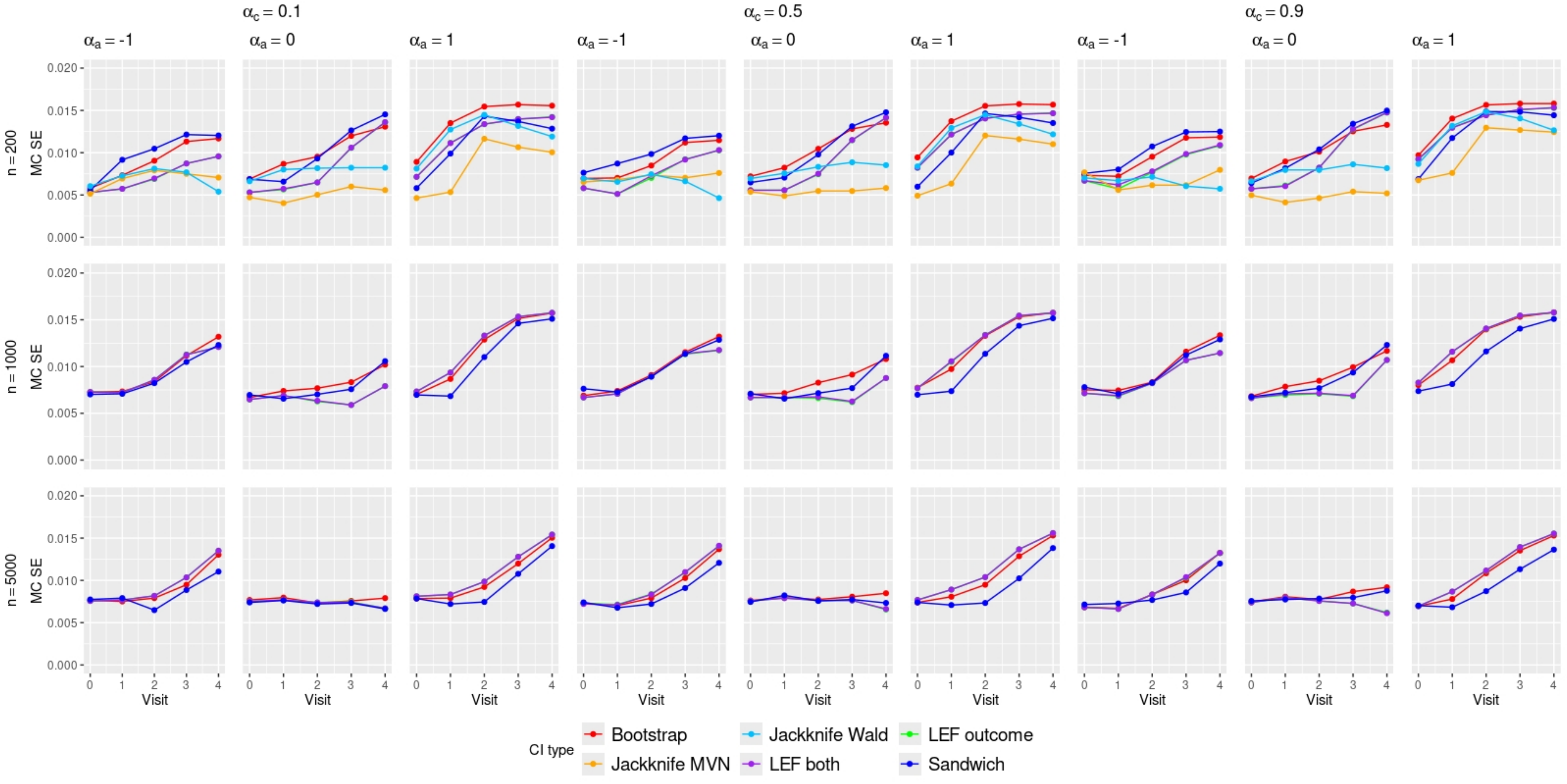

Figure 21: Monte Carlo standard error the CI coverage under **high event rates**. Bootstrap: CIs constructed by nonparametric bootstrap; LEF both: CIs constructed by applying Approach 2 of LEF bootstrap; LEF outcome: CIs constructed by applying Approach 1 of LEF bootstrap; Jackknife Wald: CIs constructed by applying Approach 1 of Jackknife resampling; Jackknife MVN: CIs constructed by applying Approach 2 of Jackknife resampling; Sandwich: CIs based on the sandwich variance estimator

5.8 Convergence issues

During the simulations, we encountered estimation errors when the `glm` function in `R` was used by the `trial_msm` function of the `TrialEmulation` package to estimate the MSM parameters in small sample size scenarios ($n = 200$). The MSM parameters $\beta_{1,3}$ and $\beta_{1,4}$ in equation (11) of the main text were often inestimable in such scenarios due to data sparsity issues. When such errors occurred, the default in the `predict.glm` function used for estimating the MRD was to treat the inestimable parameters as equal to 0. Therefore, we are still able to estimate the MRD and construct nonparametric bootstrap CIs, LEF bootstrap CIs and Jackknife Wald CIs. However, the data sparsity issues also led to difficulty to construct Sandwich CIs because of non-positive definiteness and non-symmetry of the variance matrices.

From Table 13, we see that, whilst we encountered little to no convergence issues when constructing nonparametric bootstrap CIs, LEF bootstrap CIs and Jackknife Wald CIs for all data scenarios, there were substantially more problems of non-positive definiteness and non-symmetry when computing the sandwich variance matrices. This led to a high rate of failures in constructing the Sandwich CIs as the multivariate normal distribution for sampling requires a symmetric, positive definite variance matrix. When the sample size was small ($n = 200$), the failure rate for Sandwich CIs was between 3% and 80% whilst the failure rate for sample size $n = 1000$ was between less than 1% and 35%. Although we have not included the failure rates by confounding strength, we note that the failure rate for the Sandwich CIs was further exacerbated in scenarios with $n = 200$ and high treatment prevalence, averaging at around between 42% and 80%. On occasion, we observed that this was due to `R` being too restrictive with the matrix properties. In some cases, the sandwich variance matrix was almost symmetric with differences between symmetric values at a negligible magnitude of 10^{-4}, so discarding the results based on such a difference and forcing a symmetric matrix for Sandwich CIs may have been judicious, and may have modified the coverage rate results for these CIs slightly. For very large sample sizes $n = 5000$, the failure rate was negligible across all CI methods.

Jackknife MVN CIs also encountered many construction issues, where for low and high treatment prevalence scenarios, this method failed for roughly half of the simulations, making it unreliable for such simulation scenarios. These construction issues were often due to the treatment coefficients at the later visits ($k = 3, 4$) in the MSM being inestimable in some jackknife samples. Therefore the jackknife variance estimate in equation (10) of the main text could not be calculated.

Table 13: Proportions of simulations with CI construction failures **stratified by outcome event rate, sample size and treatment prevalence and averaged over confounding strength scenarios**. Bootstrap: CIs constructed by nonparametric bootstrap; LEF both: CIs constructed by applying Approach 2 of LEF bootstrap; LEF outcome: CIs constructed by applying Approach 1 of LEF bootstrap; Jackknife Wald: CIs constructed by applying Approach 1 of Jackknife resampling; Jackknife MVN: CIs constructed by applying Approach 2 of Jackknife resampling; Sandwich: CIs based on the sandwich variance estimator

Outcome event rate	Sample size	Treatment prevalence	Bootstrap	LEF outcome	LEF both	Sandwich	Jackknife MVN	Jackknife Wald
Low	200	-1	0.00	0.00	0.00	0.06	0.47	0.00
		0	0.00	0.00	0.00	0.28	0.04	0.00
		1	0.00	0.00	0.00	0.80	0.49	0.00
	1000	-1	0.00	0.00	0.00	0.00		
		0	0.00	0.00	0.00	0.04		
		1	0.00	0.00	0.00	0.35		
	5000	-1	0.00	0.00	0.00	0.00		
		0	0.00	0.00	0.00	0.00		
		1	0.00	0.00	0.00	0.01		
Medium	200	-1	0.00	0.00	0.00	0.04	0.47	0.00
		0	0.00	0.00	0.00	0.12	0.05	0.00
		1	0.00	0.00	0.00	0.62	0.48	0.00
	1000	-1	0.00	0.00	0.00	0.00		
		0	0.00	0.00	0.00	0.01		
		1	0.00	0.00	0.00	0.09		
	5000	-1	0.00	0.00	0.00	0.00		
		0	0.00	0.00	0.00	0.00		
		1	0.00	0.00	0.00	0.00		
High	200	-1	0.00	0.00	0.00	0.03	0.45	0.00
		0	0.00	0.00	0.00	0.07	0.08	0.00
		1	0.00	0.00	0.00	0.42	0.48	0.00
	1000	-1	0.00	0.00	0.00	0.00		
		0	0.00	0.00	0.00	0.00		
		1	0.00	0.00	0.00	0.03		
	5000	-1	0.00	0.00	0.00	0.00		
		0	0.00	0.00	0.00	0.00		
		1	0.00	0.00	0.00	0.00		

6 Target trial protocol to estimate the per-protocol effect of HAART on all-cause mortality using the HERS data

Table 14: Protocol of a target trial to estimate the per-protocol effect of HAART on all-cause mortality

Eligibility criteria	HIV-infected women with no prior history of HAART.
Treatment strategies	Patients in the treatment arm will receive HAART throughout the trial. Patients in the control arm will not receive HAART during the trial (but may take other antiviral treatments).
Assignment procedures	Patients will be randomly assigned to either treatment strategy at baseline and will be aware of the strategy to which they have been assigned.
Follow-up period	Patients are followed from treatment randomisation at baseline visit, for a course of 5 visits spaced 6 months apart, or until event or loss to follow-up, whichever occurs first.
Outcome	Death from any cause
Causal contrast of interest	Per-protocol effect of sustained HAART treatment on all-cause mortality
Statistical Methods	Patients' follow-up will be artificially censored at the time they deviate from their assigned treatment strategy at baseline. IPW using time-varying covariates will be used to address the selection bias caused by this artificial censoring and dependent censoring due to patient's loss to follow-up.

7 Summary statistics of the estimated inverse probability of treatment and censoring weights by treatment arms in the HERS data analysis

Table 15 presents the summary statistics of the estimated inverse probability of treatment and censoring weights by treatment arms in the HERS data analysis, which suggests no practical violation of the positivity assumption of treatment adherence and censoring in the HERS data.

Table 15: Summary statistics of estimated inverse probability of treatment and censoring weights by treatment arms in the HERS data analysis

Assigned treatment	Minimum	1st quantile	Mean	Median	3rd quantile	Maximum
1	0.52	0.91	1.01	1.00	1.01	2.47
0	0.50	0.94	1.00	1.00	1.00	2.53

References

[1] Danaei G, Rodríguez LAG, Cantero OF et al. Observational data for comparative effectiveness research: An emulation of randomised trials of statins and primary prevention of coronary heart disease. *Statistical Methods in Medical Research* 2013; 22(1): 70–96. doi:10.1177/0962280211403603.

[2] Daniel R, Cousens S, De Stavola B et al. Methods for dealing with time-dependent confounding. *Statistics in Medicine* 2013; 32(9): 1584–1618. doi:10.1002/sim.5686.

[3] Murray EJ, Caniglia EC and Petito LC. Causal survival analysis: A guide to estimating intention-to-treat and per-protocol effects from randomized clinical trials with non-adherence. *Research Methods in Medicine & Health Sciences* 2021; 2(1): 39–49. doi:10.1177/2632084320961043.

[4] Robins JM, Hernán MA and Brumback B. Marginal Structural Models and Causal Inference in Epidemiology. *Epidemiology* 2000; 11(5): 550–560. doi:10.1097/00001648-200009000-00011.

[5] Morris TP, White IR and Crowther MJ. Using simulation studies to evaluate statistical methods. *Statistics in Medicine* 2019; 38(11): 2074–2102. doi:10.1002/sim.8086.
